# Associations of lipoprotein particle profile and objectively measured physical activity and sedentary time in schoolchildren: a prospective cohort study

**DOI:** 10.1186/s12966-022-01244-w

**Published:** 2022-01-21

**Authors:** Paul Remy Jones, Tarja Rajalahti, Geir Kåre Resaland, Eivind Aadland, Jostein Steene-Johannessen, Sigmund Alfred Anderssen, Tone Frost Bathen, Trygve Andreassen, Olav Martin Kvalheim, Ulf Ekelund

**Affiliations:** 1 Department of Sports Medicine, Norwegian School of Sport Sciences, Ullevål Stadion, Post Box 4014, 0806 Oslo, Norway; 2 Department of Chemistry, University of Bergen, Bergen, Norway; 3 Førde Health Trust, Førde, Norway; 4 Center for Physically Active Learning, Faculty of Education, Arts and Sports, Campus Sogndal, Western Norway University of Applied Sciences, Sogndal, Norway; 5 Department of Sport, Food and Natural Sciences, Western Norway University of Applied Sciences, Sogndal, Norway; 6 Department of Circulation and Medical Imaging, NTNU — Norwegian University of Science and Technology, Trondheim, Norway; 7 MR Core Facility, Department of Circulation and Medical Imaging, NTNU — Norwegian University of Science and Technology, Trondheim, Norway

**Keywords:** Epidemiology, Lipoproteins, Metabolism, Physical activity, Sedentary time

## Abstract

**Background:**

Our understanding of the mechanisms through which physical activity might benefit lipoprotein metabolism is inadequate. Here we characterise the continuous associations between physical activity of different intensities, sedentary time, and a comprehensive lipoprotein particle profile.

**Methods:**

Our cohort included 762 fifth grade (mean [SD] age = 10.0 [0.3] y) Norwegian schoolchildren (49.6% girls) measured on two separate occasions across one school year. We used targeted proton nuclear magnetic resonance (^1^H NMR) spectroscopy to produce 57 lipoprotein measures from fasted blood serum samples. The children wore accelerometers for seven consecutive days to record time spent in light-, moderate-, and vigorous-intensity physical activity, and sedentary time. We used separate multivariable linear regression models to analyse associations between the device-measured activity variables—modelled both prospectively (baseline value) and as change scores (follow-up minus baseline value)—and each lipoprotein measure at follow-up.

**Results:**

Higher baseline levels of moderate-intensity and vigorous-intensity physical activity were associated with a favourable lipoprotein particle profile at follow-up. The strongest associations were with the larger subclasses of triglyceride-rich lipoproteins. Sedentary time was associated with an unfavourable lipoprotein particle profile, the pattern of associations being the inverse of those in the moderate-intensity and vigorous-intensity physical activity analyses. The associations with light-intensity physical activity were more modest; those of the change models were weak.

**Conclusion:**

We provide evidence of a prospective association between time spent active or sedentary and lipoprotein metabolism in schoolchildren. Change in activity levels across the school year is of limited influence in our young, healthy cohort.

**Trial registration:**

ClinicalTrials.gov, #NCT02132494. Registered 7th April 2014

**Supplementary Information:**

The online version contains supplementary material available at 10.1186/s12966-022-01244-w.

## Background

Cardiovascular diseases (CVDs) are the leading cause of death globally [1]. The associations between physical activity (PA), CVD incidence and mortality are well-established, and raising levels of PA is considered a cornerstone of disease prevention for both individuals and populations [2–4]. In children and adolescents, higher levels of PA are associated with better composite scores of cardiometabolic health [5, 6]. However, the relationship with individual cardiometabolic risk factors, such as blood lipids, is inconsistent and most data are from cross-sectional studies [7]. It is also unclear whether these associations are independent of adiposity [8].

More detailed metabolic phenotyping—metabolomics—can improve our understanding of the mechanisms by which PA benefits metabolism by providing detailed information of the molecules and pathways involved [9]. A small number of studies have revealed associations of PA with a number of novel measures of lipid metabolism not observable with the standard lipid profile [10, 11]. We have previously examined the theoretical effects of replacing time spent sedentary with moderate- to vigorous-intensity physical activity (MVPA) using isotemporal substitution, but were limited in our ability to examine temporality of the associations due to the cross-sectional design [12].

In this study, we used targeted proton nuclear magnetic resonance (^1^H NMR) spectroscopy to produce comprehensive lipoprotein particle profiles for our cohort of healthy schoolchildren, then examined the continuous associations with objectively measured PA of different intensities and sedentary time over one school year. We also explored potential confounding of associations by adiposity.

## Methods

Additional information regarding blood sample handling and the ^1^H NMR protocol are reported in the Supplementary Material.

### Sample population

We drew our cohort from children who participated in the Active Smarter Kids (ASK) study; a cluster randomised controlled trial (RCT) in which the effect of a school-based PA intervention on academic performance was investigated (https://clinicaltrials.gov, #NCT02132494) [13]. Of the 60 schools approached, 57 (1129 children) participated. The PA intervention was delivered over one academic year. Baseline testing took place in 2014. Changes in physical activity levels were of a similar degree for children who either received the intervention or did not [13]. We therefore pooled all children for this analysis.

### Ethics

The Regional Committee for Medical Research Ethics approved the study protocol (2013/1893). Written consent was obtained from each child’s parent(s) or legal guardian(s) and from school authorities prior to testing. Procedures and methods abide by the World Medical Association’s Declaration of Helsinki [14].

### Exposure variables

The children wore triaxial accelerometers (ActiGraph GT3X+, ActiGraph LLC, Pensacola, FL) positioned on their right hip for seven consecutive days, but not during sleep or water-based activities. Children with at least four valid days of accelerometer measurements were included in the analytical sample. We considered a valid day ≥480 min of monitor wear time between 0600 and 0000. This combination of valid days and minimum daily wear time per day has been shown to provide reliable estimates of physical activity measured by ActiGraph accelerometers in children [15]. Non-wear time was defined as ≥20 min of zero counts [16]. The accelerometer data were processed using commercially available KineSoft software (version 3.3.80, KineSoft, Loughborough, United Kingdom) and 10-s epochs. We classified PA intensity and sedentary time using the Evenson cut points of count data: sedentary time (≤100 counts·min^–1^), low-intensity physical activity (LPA; >100 and <2296 counts·min^–1^), moderate-intensity physical activity (MPA; ≥2296 and <4012 counts·min^–1^), and vigorous-intensity physical activity (VPA; ≥4012 counts·min^–1^) [17, 18].

### Outcome variables

The children fasted overnight, and a trained nurse or phlebotomist drew blood serum samples between 0800 and 1000. NMR spectra were recorded on a Bruker Avance III 600 MHz spectrometer (Bruker BioSpin GmbH, Karlsruhe, Germany). We selected the lipoprotein NMR spectral regions quantitatively associated to lipoprotein concentrations as explanatory variables to partial least squares (PLS) modelling. The PLS model response variables were determined by high-performance liquid chromatography (HPLC) [19, 20]. In total, 106 serum samples were randomly selected for both HPLC and NMR analysis. We used a Monte Carlo resampling approach to calculate individual PLS models with optimal prediction ability for the HPLC data [21]. Lipoprotein particle numbers for all samples were predicted from these models, and the 20 lipoprotein subclasses were reduced to 15 [22]. Due to the elution of lipid-poor pre-β_1_ high-density lipoproteins (HDLs), the “spherical particle model” for calculating particle number cannot be applied to the HDL7 minor subclass [20, 23]. Hence, the particle number of the HDL VS subclass in our study was calculated using HDL6 only. Lipoprotein measures available for subsequent analysis comprised: total serum cholesterol concentration; total triglycerides concentration; non-HDL cholesterol concentration (calculated by subtracting HDL cholesterol concentration from the total cholesterol concentration); particle number, cholesterol concentration and triglycerides concentration of 15 lipoprotein subclasses; and average particle diameter of very low-density lipoprotein (VLDL), low-density lipoprotein (LDL), and HDL particles. Though intact chylomicron particles cannot be distinguished from the largest VLDL particles using NMR spectroscopy, the nomenclature from the HPLC method, which does distinguish the two, was retained. For consistency, the chylomicron subclass was not incorporated when calculating measures of the VLDL class: VLDL cholesterol concentration, VLDL triglycerides concentration, or average VLDL particle size. However, given that the blood samples were drawn subsequent to an overnight fast, it is unlikely that the particles labelled chylomicrons are of intestinal origin and should therefore be considered very large VLDLs [24].

### Anthropometrics

We measured body weight to the nearest 0.1 kg using an electronic scale (Seca 899, SECA GmbH, Hamburg, Germany). Height—with shoes removed, facing forwards—to the nearest 0.1 cm using a stadiometer (Seca 217, SECA GmbH, Hamburg, Germany). We calculated body mass index (BMI) as weight divided by height squared (kg·m^–2^). Using a measuring tape (Seca 201, SECA GmbH, Hamburg, Germany), we took two measurements of waist circumference—between the lowest palpable rib and iliac crest, the child having gently exhaled—to the nearest 0.1 cm. If the two measurements differed by more than 1.0 cm a third was taken; the mean of the two with the least difference was used for analysis. The proportions of overweight or obese girls and boys were calculated using the International Obesity Task Force’s (IOTF) sex-specific BMI cut-offs, rounding down the children’s ages at the time of testing to the nearest half-year [25].

### Sexual maturity

Each child assessed their sexual maturity against a standard set of colour images and accompanying text descriptions that corresponded to the Tanner staging method [26]. The assessments took place in a private room and the children were accompanied by a researcher of the same sex to ensure their comfort. Low frequencies of children in Tanner categories 3, 4, and 5 (*n* = 66, 5, 2, respectively of 1081 children with valid baseline data) were recorded and therefore combined into one category (≥3).

### Educational attainment of parent(s) or guardian(s)

This was considered as the highest level of attainment of a child’s mother, father, or guardian, whichever was higher. Parent(s) or guardian(s) individually completed a custom self-report study questionnaire, selecting their level of educational attainment as one of six categories. Of the six, low frequencies were recorded in the lower four categories (*n* = 4, 15, 193, 137, for categories 1–4, respectively of 1069 children with valid baseline data), so were combined into one category—*Upper secondary school*—for analysis.

### Statistical approach

We examined the prospective associations between the mean daily time spent in different intensities of activity for each of the four activity variables (LPA, MPA, VPA, and sedentary time) measured at baseline and the 57 lipoprotein variables measured at follow-up using separate linear models. Each model was adjusted for sex and parent’s/guardian’s education, and the baseline values of the respective lipoprotein measure, mean daily accelerometer wear time, age, and sexual maturity. To examine the associations with change in mean daily time spent in different intensities of activity over the follow-up period, change scores were used (follow-up minus baseline). Accelerometer wear time was also modelled as a change score, and baseline values used for the other covariates. We repeated each analysis additionally including baseline waist circumference in the model to investigate potential confounding by adiposity. For each analysis, children with valid data for all model variables were included.

All activity and lipoprotein variables were converted to *z*-scores (mean = 0.0; standard deviation [SD] = 1.0), hence the regression coefficients represent the SD unit change in lipoprotein measure for a 1 SD increase in activity variable. Changes in mean daily time spent in different intensities of activity were modelled as the *z*-score of the change (follow-up minus baseline). To account for potential within-cluster correlation and to obviate the need to transform skewed outcome variables, cluster and heteroscedasticity robust standard errors were calculated, clustered on school. We used principal component analysis (PCA) to estimate the effective number of independent tests to use for multiple testing correction. The assumption of this approach is that the independence of principal components and degrees of freedom between the original lipoprotein measures are equivalent, and that dividing the alpha value by the number of principal components that explain >95% of variance will produce only a small chance of false positives [27–29]. Using *z*-scores of the 57 lipoprotein measures, we calculated that the first five principal components explained >95% of the variance. Hence, our Bonferroni-corrected threshold for assessing associations is 0.05/5 = 0.01 (i.e., *p* <0.01). All analyses were conducted using R version 3.6.3 (R Foundation for Statistical Computing, Vienna, Austria). In addition to base R functions, we used a variety of packages within the **tidyverse** (1.3.0) suite for data manipulation. We performed the PCA analysis with **factoextra** (1.0.6) and the linear regression analysis using the **estimatr** (0.22.0) package, specifically the **lm_robust()** function. We plotted the results with **ggplot2** (3.3.0) and the custom visualisation functions **geom_stripes()** and **facet_col()** available in the **ggforestplot** (0.0.2) and **ggforce** (0.3.1) packages, respectively.

## Results

### Sample characteristics

**Fig. 1:**
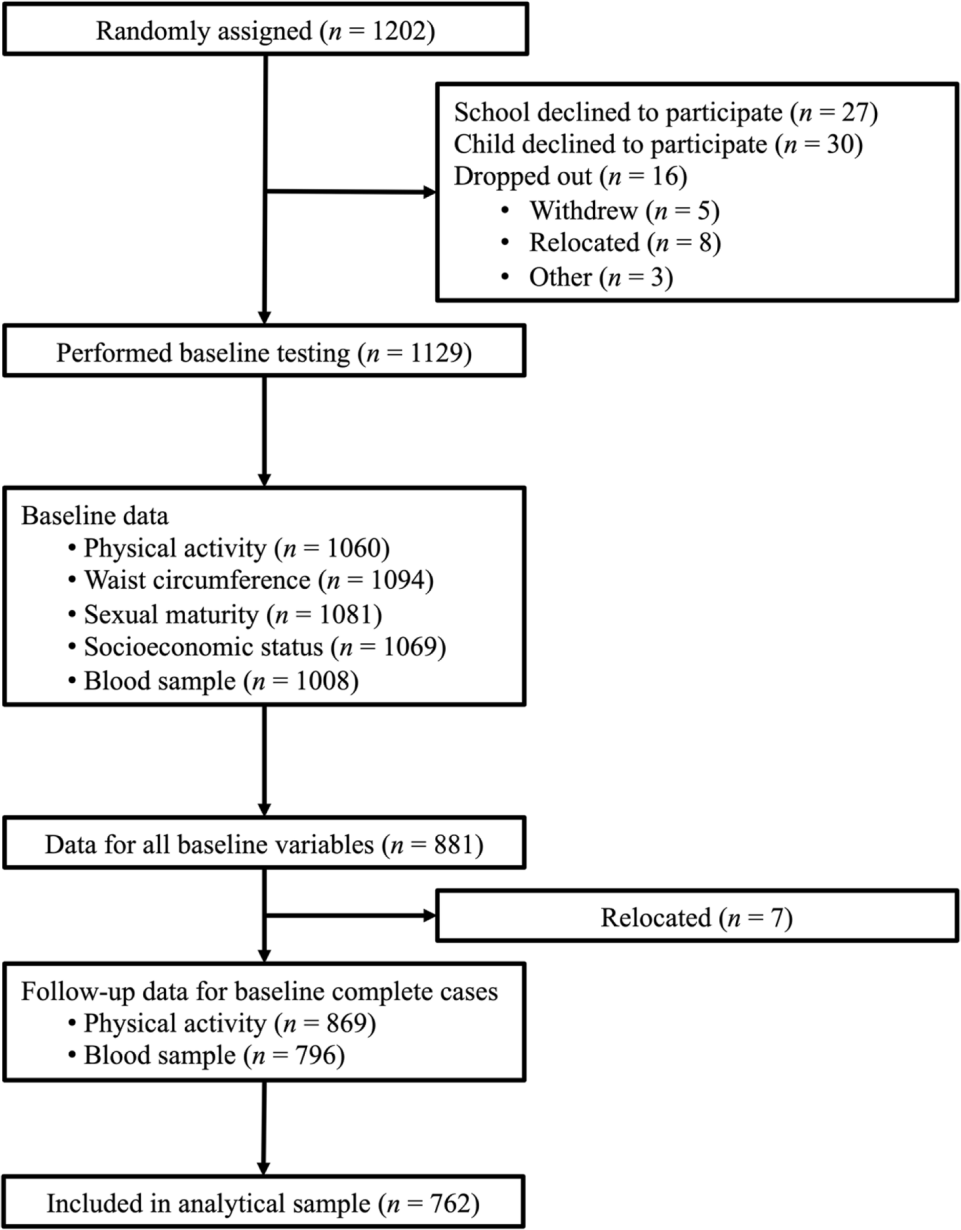
Flow of participants through the study indicating number of children that had valid data available. The final analytical sample included those children that had valid data for all baseline variables and blood samples at follow-up

Our analytical sample for the prospective analyses comprised 762 children (49.6% girls) with complete data and at least four valid days of accelerometer measurements (Fig. 1). There were 403 children (52.9%) who had seven valid days of accelerometer data. Of these 762 children, 720 had at least four days of valid follow-up accelerometer data hence were included in the change score analyses. The average interval between baseline and follow-up accelerometer testing was 46.6 weeks. Correlations between the baseline and follow-up activity intensity measures in the change analysis were moderate (Pearson’s *r* = 0.52 for VPA, 0.53 for MPA, 0.57 for LPA, and 0.53 for sedentary time; *p* <0.001 for each). Descriptive information for the analytical sample is given in Table 1. Means and SDs for the NMR lipoprotein measures are provided in Supplementary Material Table 1.

**Table 1:** Characteristics of children included in the analytical sample

Characteristic	*n* (%)	Mean (SD)
Baseline	762	
Age (years)		10.0 (0.3)
Sex		
Girls	378 (49.6)	
Boys	384 (50.4)	
Anthropometry		
Height (m)		143.1 (6.7)
Weight (kg)		37.2 (8.1)
BMI (kg·m^–2^)		18.1 (3.0)
≥25	189 (24.8)	
≥30	40 (5.2)	
Waist circumference (cm)		62.1 (7.6)
Parents’ education		
Upper secondary school	241 (31.6)	
<4 years college/university	229 (30.1)	
≥4 years college/university	292 (38.3)	
Tanner stage		
Stage 1	417 (54.7)	
Stage 2	297 (39.0)	
Stage ≥3	48 (6.3)	
Physical activity		
VPA (min·d^–1^)		32.2 (16.2)
MPA (min·d^–1^)		45.1 (12.8)
LPA (min·d^–1^)		235.2 (36.7)
SED (min·d^–1^)		466.5 (57.5)
MVPA ≥60 (min·d^–1^)	563 (73.9)	
Lipid profile^a^		
TC (mmol·L^–1^)		4.5 (0.7)
LDL-C (mmol·L^–1^)		2.5 (0.6)
HDL-C (mmol·L^–1^)		1.6 (0.3)
TG (mmol·L^–1^)^b^		0.7 [0.5, 0.9]
Follow-up	720	
Physical activity		
ΔVPA_t2–t1_ (min·d^–1^)		–4.7 (14.9)
ΔMPA_t2–t1_ (min·d^–1^)		–4.9 (12.1)
ΔLPA_t2–t1_ (min·d^–1^)		–14.3 (33.3)
ΔSED_t2–t1_ (min·d^–1^)		28.3 (52.8)
MVPA ≥60 (min·d^–1^)	451 (62.6)	
Lipid profile^a^		
TC (mmol·L^–1^)		4.5 (0.6)
LDL-C (mmol·L^–1^)		2.6 (0.6)
HDL-C (mmol·L^–1^)		1.6 (0.3)
TG (mmol·L^–1^)^b^		0.6 [0.5, 0.9]

*Abbreviations*: *BMI* body mass index, *HDL-C* high-density lipoprotein cholesterol, *IQR* interquartile range, *LDL-C* low-density lipoprotein cholesterol, *LPA* light-intensity physical activity, *MPA* moderate-intensity physical activity, *MVPA* moderate- to vigorous-intensity physical activity, *SD* standard deviation, *SED* sedentary time, *TC* total cholesterol, *TG* triglycerides, *VPA* vigorous-intensity physical activity

^a^Measured using clinical chemistry. LDL-C estimated using the Friedewald formula

^b^Median [IQR]

The 367 children not included in the prospective analyses tended to be slightly older and shorter (Supplementary Material Table 2).

### Vigorous-intensity physical activity

**Fig. 2:**
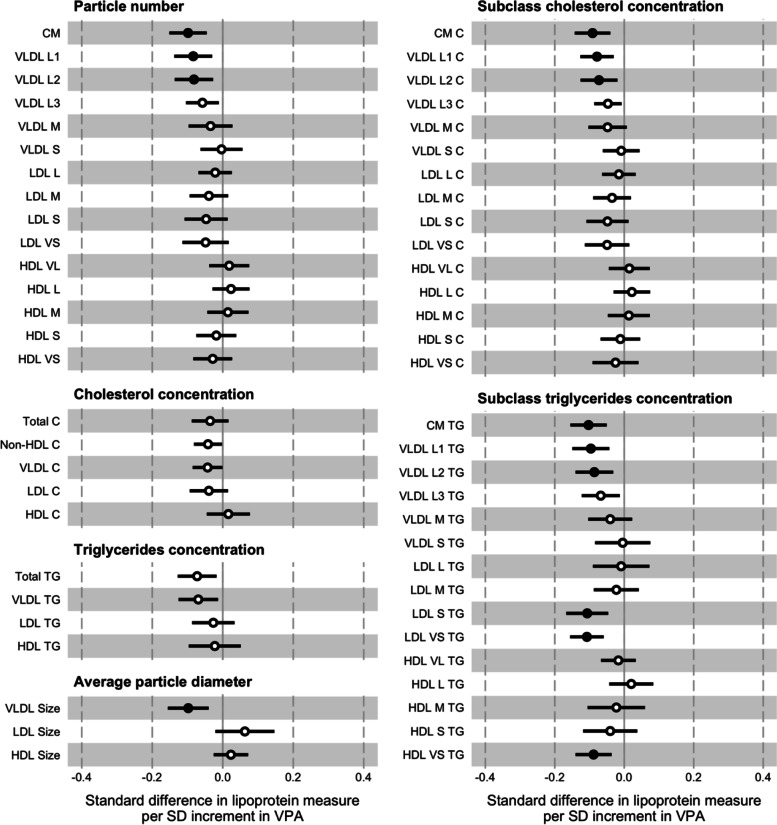
Associations between baseline vigorous-intensity physical activity (VPA) and follow-up lipoprotein measures. The association magnitudes are the standardised unit difference in lipoprotein measure per SD unit increment in activity. The models are adjusted for baseline values of accelerometer wear time, age, lipoprotein measure, parents’ education, sex, sexual maturity, and waist circumference. Cluster-robust standard errors were calculated, clustered on the school variable. Filled circles are *p* <0.01. Error bars are 95% confidence intervals. Abbreviations: CM = chylomicron; HDL = high-density lipoprotein; LDL = low-density lipoprotein; SD = standard deviation; VLDL = very low-density lipoprotein; -C = cholesterol; -L = large; -M = medium; -S = small; -TG = triglycerides; -VL = very large; -VS = very small

In the prospective analysis, there were inverse associations between a 1 SD increment in VPA (16.2 min·d^–1^) and all measures of the VLDL particles (Fig. 2; Supplementary Material Table 3). For the individual measures of particle number, cholesterol concentration, and triglycerides concentration the effect sizes decreased from the largest to smallest of these particles (e.g., –1.32 x 10^–1^ nmol·L^–1^ or –0.13 SD; 95% CI = –0.19, –0.06; *p* <0.001 for VLDL L1 particle number). The associations between VPA and all but one of the LDL measures were also inverse, though the effect sizes typically smaller than for the measures of larger ApoB-containing particles. The triglycerides concentrations of the two subclasses of the smallest LDL particles were an exception. For the HDL measures, the directions of associations tended to differ dependent on subclass. The effect sizes were mostly modest, though larger for the triglycerides concentrations of the two subclasses of the smallest HDL particles. The association with the average diameter of VLDL particles was inverse and effect size larger than those of the positive associations with the average diameter of LDL and HDL particles. Regarding the more traditional lipid measures, higher VPA was inversely associated with TC, non-HDL-C, and LDL-C concentrations, and positively associated with HDL-C concentration. The effect sizes were modest. In contrast, the effect size was larger for the inverse association with total triglycerides concentration. The pattern of associations remained broadly similar having included waist circumference as an additional covariate in the models. The degree of attenuation for individual measures ranged from small to moderate and tended to be greater for the subclasses of larger particles (–8.60 x 10^–2^ nmol·L^–1^ or –0.08 SD; 95% CI = –0.14, –0.03; *p* <0.01 for VLDL L1 particle number).

The associations with a 1 SD change in VPA (14.9 min·d^–1^) between baseline and follow-up measurement occasions were weak (Supplementary Material Fig. 1; Supplementary Material Table 7). Adjustment for waist circumference had a negligible effect on these associations.

### Moderate-intensity physical activity

**Fig. 3:**
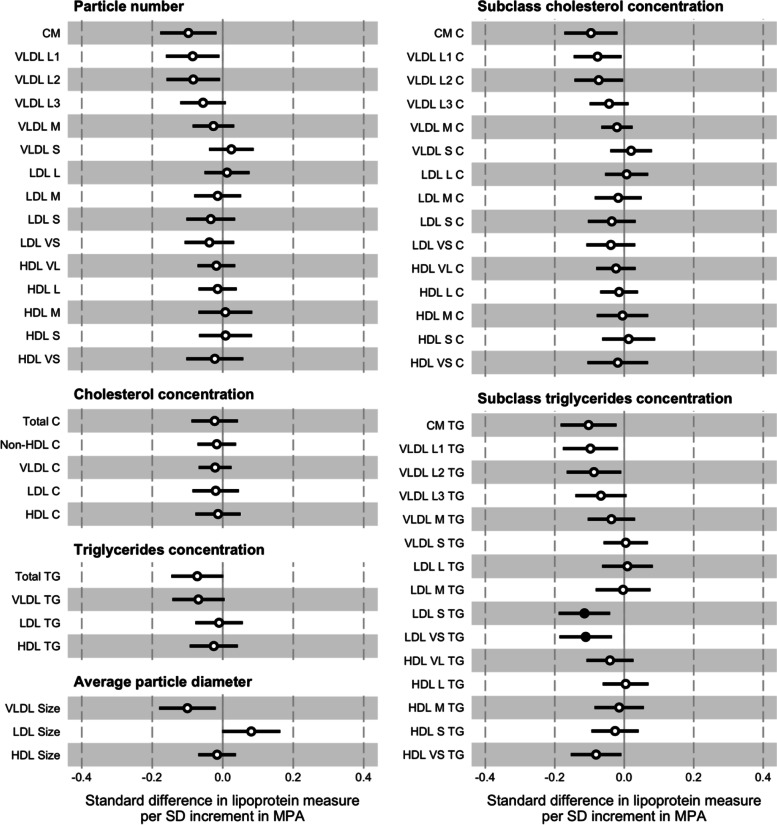
Associations between baseline moderate-intensity physical activity (MPA) and follow-up lipoprotein measures. The association magnitudes are the standardised unit difference in lipoprotein measure per SD unit increment in activity. The models are adjusted for baseline values of accelerometer wear time, age, lipoprotein measure, parents’ education, sex, sexual maturity, and waist circumference. Cluster-robust standard errors were calculated, clustered on the school variable. Filled circles are *p* <0.01. Error bars are 95% confidence intervals. Abbreviations: CM = chylomicron; HDL = high-density lipoprotein; LDL = low-density lipoprotein; SD = standard deviation; VLDL = very low-density lipoprotein; -C = cholesterol; -L = large; -M = medium; -S = small; -TG = triglycerides; -VL = very large; -VS = very small

In the prospective analysis, the pattern of associations between a 1 SD increment in MPA (12.8 min·d^–1^) and the lipoprotein measures was broadly similar to that of a 1 SD increment in VPA (Fig. 3; Supplementary Material Table 4). The effect sizes were smaller for all but four of the 57 individual measures (e.g., –1.14 x 10^–1^ nmol·L^–1^ or –0.11 SD; 95% CI = –0.19, –0.03; *p* <0.01 for VLDL L1 particle number). Many of the coefficients were close to null. Including waist circumference as an additional covariate in the models had a negligible effect.

Generally, the pattern of associations with a 1 SD change in MPA (12.1 min·d^–1^) was similar to those in the VPA change model, though the effect sizes for many individual measures were larger (Supplementary Material Fig. 2; Supplementary Material Table 8). Adjustment for waist circumference had a negligible effect on these associations.

### Light-intensity physical activity

**Fig. 4:**
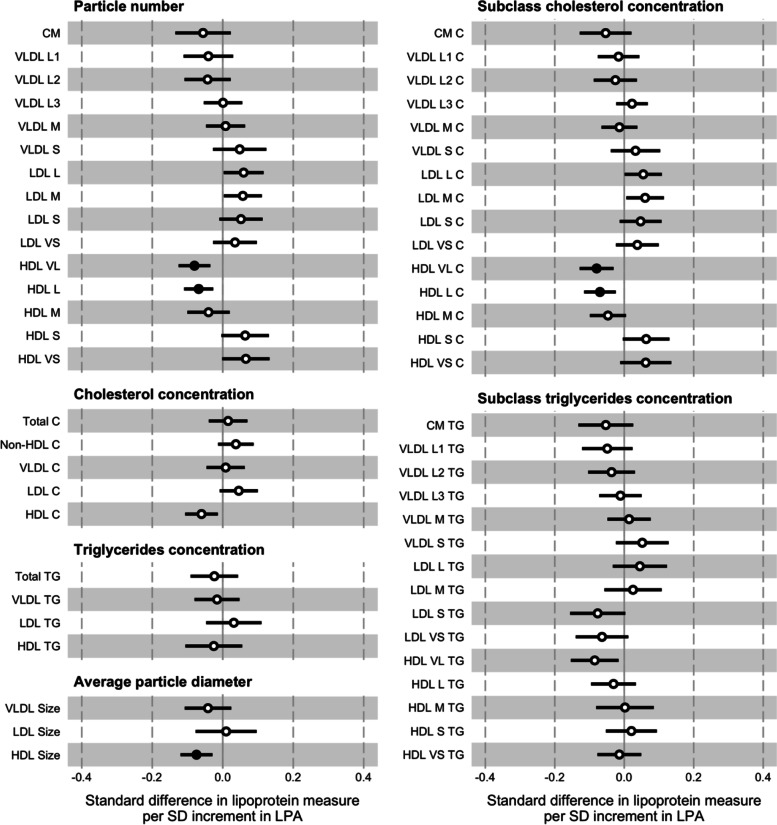
Associations between baseline light-intensity physical activity (LPA) and follow-up lipoprotein measures. The association magnitudes are the standardised unit difference in lipoprotein measure per SD unit increment in activity. The models are adjusted for baseline values of accelerometer wear time, age, lipoprotein measure, parents’ education, sex, sexual maturity, and waist circumference. Cluster-robust standard errors were calculated, clustered on the school variable. Filled circles are *p* <0.01. Error bars are 95% confidence intervals. Abbreviations: CM = chylomicron; HDL = high-density lipoprotein; LDL = low-density lipoprotein; SD = standard deviation; VLDL = very low-density lipoprotein; -C = cholesterol; -L = large; -M = medium; -S = small; -TG = triglycerides; -VL = very large; -VS = very small

In contrast to the VPA and MPA analyses, the associations between a 1 SD increment in LPA (36.7 min·d^–1^) and the VLDL measures tended to be more modest (Fig. 4; Supplementary Material Table 5). The effect sizes with certain individual subclasses were small, and those with the VLDL L3 and VLDL M subclasses almost null (e.g., –4.53 x 10^–2^ nmol·L^–1^ or –0.04 SD; 95% CI = –0.12, 0.03; *p* = 0.24 for VLDL L1 particle number). In contrast to the VPA and MPA analyses, the directions of associations with the LDL subclass measures tended to be positive, though the distinct inverse associations with the triglycerides concentrations of the LDL S and LDL VS subclasses were replicated. The divergent directions of associations between the particle numbers and cholesterol concentrations of the HDL subclasses were apparent, but in the opposite directions to those in the VPA analysis. The effect sizes for these measures tended to be larger than in the VPA analysis, though not for the subclass triglycerides concentrations. Including waist circumference as an additional covariate in the models had a limited effect.

The associations between a 1 SD change in LPA (33.3 min·d^–1^) and the lipoprotein particle profile tended to be weak (Supplementary Material Fig. 3; Supplementary Material Table 9). Adjustment for waist circumference had a negligible effect on these associations.

### Sedentary time

**Fig. 5:**
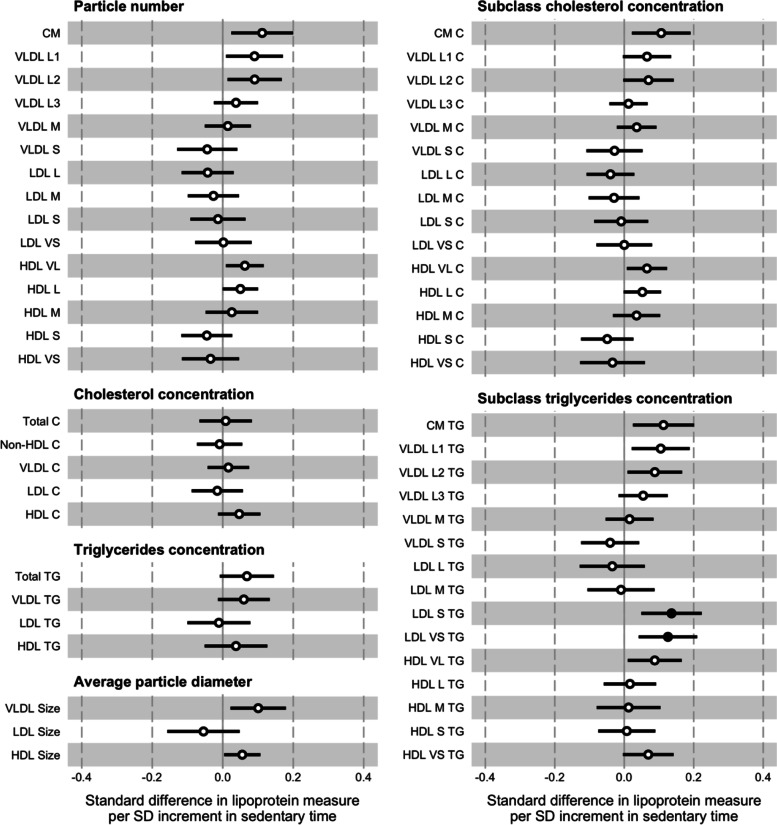
Associations between baseline sedentary time and follow-up lipoprotein measures. The association magnitudes are the standardised unit difference in lipoprotein measure per SD unit increment in activity. The models are adjusted for baseline values of accelerometer wear time, age, lipoprotein measure, parents’ education, sex, sexual maturity, and waist circumference. Cluster-robust standard errors were calculated, clustered on the school variable. Filled circles are *p* <0.01. Error bars are 95% confidence intervals. Abbreviations: CM = chylomicron; HDL = high-density lipoprotein; LDL = low-density lipoprotein; SD = standard deviation; VLDL = very low-density lipoprotein; -C = cholesterol; -L = large; -M = medium; -S = small; -TG = triglycerides; -VL = very large; -VS = very small

The associations between a 1 SD increment in sedentary time (57.5 min·d^–1^) were typically stronger with the VLDL particle measures (Fig. 5; Supplementary Material Table 6). The directions of associations with all but one of these measures were the opposite of those in the prospective analysis of MPA and all but the VLDL S subclass in the VPA analysis (e.g., 1.22 x 10^–1^ nmol·L^–1^ or 0.12 SD; CI = 0.03, 0.21; *p* = 0.01 for VLDL L1 particle number). Though the effect sizes were smaller, the pattern of effect sizes decreasing from the largest to the smallest particles was replicated. For the HDL subclasses, the directions of associations with measures of particle numbers and cholesterol concentrations tended to differ dependent on the particle size, though the majority of individual effects were small to medium. The effect sizes for the triglycerides concentrations of the two subclasses of the smallest LDL particles were again more pronounced compared to other LDL subclass measures, which were typically small to null. The effect sizes of the associations for the triglycerides concentrations of the subclasses of the largest and smallest HDL particles were relatively large compared to the other HDL subclasses. The degree of attenuation for individual measures having included waist circumference in the model were generally small.

The directions of association between a 1 SD change in sedentary time (52.8 min·d^–1^) and the lipoprotein measures tended to be the opposite of those in the VPA and MPA change models, and the effect sizes smaller (Supplementary Material Fig. 4; Supplementary Material Table 10). Adjustment for waist circumference had a negligible effect on these associations.

## Discussion

We aimed to investigate the prospective associations of objectively measured PA and sedentary time with a comprehensive lipoprotein particle profile across the school year in fifth grade schoolchildren. We found that, broadly speaking, higher levels and higher intensities of PA are associated with an apparently favourable profile, whereas greater time spent sedentary seems detrimental. Effect sizes were modest, however, which suggests that the influence of PA on individual measures is limited. Associations with changes in PA or sedentary time were weak, which may potentially be due to the small overall changes in this active population.

Previous studies have reported similar beneficial lipoprotein particle profiles with higher levels of PA. In a study of device-measured physical activity and a comprehensive metabolic profile in adolescents, the authors showed a number of beneficial associations with MVPA, such as inverse associations with the triglycerides concentrations of LDL and VLDL subclasses, that were comparatively more robust than and tended to be in the opposite direction to those with sedentary time [10]. Associations between the metabolic profile and 3-year change in activity were generally weak. In another study, metabolic profiles of adults who self-reported their level of PA as “active” were compared to those who reported being “inactive” on two occasions at least five years apart [11]. Being consistently active was inversely associated with the particle concentrations of all ApoB-containing lipoprotein subclasses, a number of measures of subclass triglycerides concentration, and positively associated with both the particle concentrations of and cholesterol concentrations of the larger HDL subclasses. Many of the associations were more pronounced compared to those in our results, which could be for several reasons. Firstly, the effect sizes reported for each lipoprotein measure were expressed using the SD unit *difference* in leisure-time PA between those categorised as active or inactive, which are likely to be greater than the SD unit *increment*s of the continuous PA intensity measures used in our study. The participants tended to be older and the time elapsed between the baseline and follow-up measurements far greater (e.g., 16 years in one of the included cohorts) than in our study. Thus, there is more likely to be greater variance in metabolism due to the accumulation of comorbidities with time and age. Also, activity levels of the included participants were likely more consistent across the two time points given that consistent level of activity was a criterion for inclusion.

Recent evidence suggests that the well-recognised causal effect of LDL on atherosclerotic CVD risk may not be a result of the lipid mass that the particles carry, but of the concentration of particles in the circulation [30–32]. Apolipoprotein B is the primary apolipoprotein of chylomicron, VLDL, and LDL particles, and likely the causal trait that enables the lipids carried by these lipoproteins to exert their influence on CVD risk [33, 34]. Moreover, all ApoB-containing lipoproteins up to 70 nm diameter, which includes triglyceride-rich and cholesterol-rich chylomicron and VLDL remnant particles, can penetrate the arterial intima and are thought to be similarly atherogenic [35]. Our results demonstrate relatively stronger associations of PA and sedentary time with VLDL particles, compared to the LDLs. Given that the number of circulating particles likely determines the probability of them entering and being retained in the intima, any cardioprotective effects of increased PA or reduced time spent sedentary seem likely expressed through the metabolism of these larger apolipoprotein B-containing subclasses [36]. Unexpectedly, LPA was positively associated with many LDL subclass measures. This contradicts existing literature, which has shown that LPA has minimal effect on LDL-C concentration and is possibly due to confounding whereby those children who spend more time in LPA spend less time in MPA or VPA [37].

Given that VLDLs are the primary carriers of triglycerides and triglycerides the primary lipid component of VLDLs, any effect of PA on VLDL particles will therefore likely be due to an effect on triglycerides metabolism. Though VLDL triglycerides appear not to play a significant role as substrate for lipid oxidation during exercise, increased rates of VLDL triglycerides clearance subsequent to a bout of aerobic exercise have been demonstrated [38, 39]. This exercise-induced reduction in circulating triglycerides concentration is typically abolished within 48 hours after the exercise bout [40]. Thus, the prospective associations of VPA and MPA with lower levels of VLDL measures in this study may reflect the acute effects of consistent repeated exercise-induced reductions in triglycerides concentration across the follow-up period, as opposed to more permanent metabolic adaptations to chronic PA behaviour. This finding supports that of the aforementioned study of adolescents in which the cross-sectional associations between objectively measured MVPA and the metabolic profile did not differ based on previous activity levels, which the authors interpreted as the effect of PA being dependent on recent engagement and consequently reversible if activity ceases [10].

Substantial structural, compositional, and functional heterogeneity exists between HDL particles. In addition to reverse cholesterol transport, HDL particles participate in antioxidative, anti-inflammatory, and anti-infectious activities, among others, and this assortment of biological functions seems to be mediated by different particle subpopulations [41]. Surprisingly, in our study particle numbers and cholesterol concentrations of the HDL subclasses were directionally consistent between VPA and sedentary time, suggesting a shift towards larger HDL particles and increased serum HDL cholesterol. However, the associations were weak in both analyses and negligible in the MPA model, so may not be true effects. If true, it is challenging to provide a mechanistic explanation for this apparent paradox contingent on energy expenditure alone, especially given that the associations were in the reverse direction and quite robust in the LPA analyses. Instead, these results may reflect the poor characterisation by particle number or lipid mass of HDL physicochemical and functional heterogeneity, and our incomplete understanding of how HDL function changes with either total PA or PA of different intensities. Historically, that higher HDL cholesterol levels are associated with higher levels of PA and lower CVD indicated a potential means through which PA exerts its cardioprotective effect [42]. However, recent evidence from Mendelian randomisation and clinical trials in which HDL cholesterol concentration was increased significantly with pharmacotherapy but failed to result in a concomitant reduction in CVD event rate compared to placebo indicate that a direct causal effect of HDL cholesterol level on CVD is unlikely [43–45]. Furthermore, there is preliminary evidence that exercise benefits some HDL attributes independent of changes in HDL cholesterol [46]. Consequently, greater research effort has been directed to quantifying HDL functionality, its influence on CVD risk, and the effects of PA on HDL beyond the traditional lipid profile [47, 48]. The generally modest associations with HDL measures in our results suggest the cardioprotective effects of PA are either inadequately characterised by measures of particle number and lipid load, or alternatively, indicative of limited metabolic perturbation in our young, healthy cohort.

In our analyses, associations were moderately attenuated having adjusted for waist circumference, which suggests an independent effect of PA and sedentary time on our lipoprotein measures. Adiposity has been shown to be causally associated with the lipoprotein particle profile in young adults, and that it mediates a proportion of the beneficial effects of MVPA on lipid measures [8, 49]. There is also robust evidence that higher levels of adiposity are causal for lower total PA, MVPA, and increased time spent sedentary in children, and these associations are much stronger when adiposity is modelled as the exposure rather than the outcome [50]. Considered in the context of these studies, our results suggest that increasing VPA or MPA could benefit the lipoprotein particle profile, but an intervention that achieves a concomitant reduction in, or prevents increases in, adiposity would likely be synergistic. Furthermore, since the effects of PA on lipoprotein metabolism are likely acute and not maintained beyond a few days, any preventative programme would require frequent, repeated engagement in PA maintained across the life course. However, given that our study is observational, discussion of designing interventions is hypothetical. Replication of our findings in larger, more diverse populations with longer follow-up and greater metabolic variance are required to determine whether the potentially protective effects of PA are causal and what their impact on health might be.

### Strengths and limitations

The use of a targeted metabolomics platform enabled us to investigate a variety of lipoprotein measures, providing a more nuanced description of the associations with PA and sedentary time than could be achieved with a standard lipid profile. We modelled the activity measures both prospectively and as change scores, adjusting for the baseline value of each respective lipoprotein measure, which enabled us to examine the temporality of associations. The blood samples were drawn with the children having fasted and at a consistent time of day, limiting potential variability due to dietary intake and daily activity. We had a high level of participant compliance with the PA assessment, though we acknowledge that one week of PA assessed at two time points may not be fully representative of behavioural variability over many months. Furthermore, substantial intraindividual variation has been reported when measuring children’s PA using accelerometers over a 1-year period such that the true regression coefficients may be underestimated by up to 50% [51]. Device-based measures of PA are less prone to the biases typical of self-report activity data, such as participant exaggeration.

There were also several limitations. Our data are observational hence we cannot exclude unmeasured confounding from biasing our effect estimates. Importantly, we did not have dietary information for our children. Nonfasted samples are considered more representative of the predominant metabolic state [24]. Our use of fasted samples precluded us from investigating the potential for PA to mitigate the postprandial rise in triglycerides and commensurate rise in circulating chylomicrons, which are thought to contribute to increased atherosclerotic risk [52]. The children that participated in the ASK study were young and the period over which they were followed short, so metabolic variance is likely limited. It would be instructive to follow them for a longer period or as they transition through adolescence into adulthood to observe whether the potentially beneficial effects of PA augment with age. Our cohort are homogenous and also highly active in comparison to the other adolescents both within Norway and globally, which likely limits the generalisability of our findings to other populations [53]. Lastly, we modelled each activity variable separately and are therefore unable to assess their independent associations with the lipoprotein particle profile.

### Conclusion

Our study shows that more time spent being physically active—especially at higher intensities—is prospectively associated with a favourable lipoprotein particle profile, whereas more time spent sedentary appears to be detrimental. These associations are largely independent of adiposity. If causal, the mechanisms that drive these benefits are likely due to alterations in triglycerides metabolism, which may explain the typically inconclusive results of PA studies that only examine the standard lipid profile.

## Supplementary Information

**Additional file 1.**

## Data Availability

The datasets used and/or analysed during the current study are available from the corresponding author on reasonable request.

## Abbreviations

^1^H NMR: Proton nuclear magnetic resonance
ASK: Active Smarter Kids
BMI: Body mass index
CVD: Cardiovascular disease
HDL: High-density lipoprotein
HPLC: High-performance liquid chromatography
IOTF: International Obesity Task Force
LDL: Low-density lipoprotein
LPA: Low-intensity physical activity
MPA: Moderate-intensity physical activity
MVPA: Moderate- to vigorous-intensity physical activity
PA: Physical activity
PCA: Principal component analysis
PLS: Partial least squares
RCT: Randomised controlled trial
SD: Standard deviation
VLDL: Very low-density lipoprotein
VPA: Vigorous-intensity physical activity
