# Supplementary material for: Associations of lipoprotein particle profile and objectively measured physical activity and sedentary time in schoolchildren: a prospective cohort study
Source: Int J Behav Nutr Phys Act. 2022 Jan 21;19:5. doi: 10.1186/s12966-022-01244-w (PMC8781389; doi:10.1186/s12966-022-01244-w)
Supplement: Supplementary file 1 — Additional file 1. [file 12966_2022_1244_MOESM1_ESM.pdf]

Supplementary Material

Supplementary methods

Blood samples

Serum was drawn from an antecubital vein and obtained according to a standard protocol consisting of the following steps: i) Blood plasma was collected in 5 ml VACUETTE Serum Gel with Activator blood collection tubes (Greiner Bio-One International GmbH, Kremsmünster, Austria). ii) The tubes were carefully inverted five times and placed vertically for coagulation. iii) After 30 minutes, the samples were centrifuged at 2000 G for ten minutes. Serum was then visually inspected for residue and centrifugation was repeated if residue was present. iv) The tubes were kept in a refrigerator at 4°C before pipetting 0.5 ml into cryo tubes. v) The cryo tubes were then stored in a freezer at –20°C for up to 2 days before finally being stored at –80°C until analysis. The frozen serum samples were thawed at room temperature for approximately one hour. Aliquots of 120 µl were carefully mixed with equal amounts of phosphate buffer in Eppendorf tubes, and transferred to 3 mm SampleJet tubes by syringe. A fill height of 4 cm was used amounting to approximately 180 µl.

^{1}H NMR protocol

Serum spectra were recorded at 310 K, using a one-dimensional NOESY (noesygppr1d) pulse sequence. A total of 32 scans were acquired, using 96k data points and 30 ppm spectral width. The spectra were processed with 0.3 Hz line broadening, automatically phase-corrected and aligned to the lactate signal at 1.32 ppm. Spectra were normalised to an ERETIC signal, functioning as an external reference. Details of the ^{1}H NMR protocol have been described previously.[1]

References

1. Jones PR, Rajalahti T, Resaland GK, Aadland E, Steene-Johannessen J, Anderssen SA, et al. Associations of physical activity and sedentary time with lipoprotein subclasses in Norwegian schoolchildren: The Active Smarter Kids (ASK) study. Atherosclerosis. 2019 Sep 1;288:186–93.

Table 1. Mean and standard deviation (SD) for each lipoprotein measure in absolute concentration units

Lipoprotein variable	Baseline
	Mean (SD)
CM PN (nmol·L^{-1})	0.240 (0.392)
VLDL L1 PN (nmol·L^{-1})	1.024 (1.252)
VLDL L2 PN (nmol·L^{-1})	4.315 (4.585)
VLDL L3 PN (nmol·L^{-1})	19.189 (10.628)
VLDL M PN (nmol·L^{-1})	29.151 (13.484)
VLDL S PN (nmol·L^{-1})	45.085 (11.105)
LDL L PN (nmol·L^{-1})	213.391 (44.846)
LDL M PN (nmol·L^{-1})	469.633 (101.065)
LDL S PN (nmol·L^{-1})	222.782 (49.953)
LDL VS PN (nmol·l^{-1})	0.176 (0.036)
HDL VL PN (nmol·L^{-1})	0.087 (0.035)
HDL L PN (nmol·L^{-1})	1639.967 (736.695)
HDL M PN (nmol·L^{-1})	4327.068 (606.213)
HDL S PN (nmol·L^{-1})	5233.284 (543.149)
HDL VS PN (nmol·L^{-1})	2674.001 (249.148)
CM C (mmol·L^{-1})	0.011 (0.014)
VLDL C (mmol·L^{-1})	0.642 (0.256)
VLDL L1 C (mmol·L^{-1})	0.020 (0.018)
VLDL L2 C (mmol·L^{-1})	0.041 (0.042)
VLDL L3 C (mmol·L^{-1})	0.205 (0.088)
VLDL M C (mmol·L^{-1})	0.164 (0.079)
VLDL S C (mmol·L^{-1})	0.208 (0.053)
LDL C (mmol·L^{-1})	2.234 (0.496)
LDL L C (mmol·L^{-1})	0.716 (0.165)
LDL M C (mmol·L^{-1})	0.968 (0.223)
LDL S C (mmol·L^{-1})	0.389 (0.093)
LDL VS C (mmol·L^{-1})	0.165 (0.035)
HDL C (mmol·L^{-1})	1.465 (0.262)
HDL VL C (mmol·L^{-1})	0.080 (0.033)
HDL L C (mmol·L^{-1})	0.332 (0.156)
HDL M C (mmol·L^{-1})	0.528 (0.085)
HDL S C (mmol·L^{-1})	0.377 (0.037)
HDL VS C (mmol·L^{-1})	0.148 (0.012)
Total C (mmol·L^{-1})	4.359 (0.673)
Non-HDL C (mmol·L^{-1})	2.894 (0.663)
CM TG (mmol·L^{-1})	0.026 (0.044)
VLDL TG (mmol·L^{-1})	0.432 (0.318)
VLDL L1 TG (mmol·L^{-1})	0.039 (0.053)
VLDL L2 TG (mmol·L^{-1})	0.096 (0.102)
VLDL L3 TG (mmol·L^{-1})	0.155 (0.113)
VLDL M TG (mmol·L^{-1})	0.096 (0.049)
VLDL S TG (mmol·L^{-1})	0.046 (0.014)
LDL TG (mmol·L^{-1})	0.192 (0.035)
LDL L TG (mmol·L^{-1})	0.076 (0.017)
LDL M TG (mmol·L^{-1})	0.076 (0.014)
LDL S TG (mmol·L^{-1})	0.029 (0.006)
LDL VS TG (mmol·L^{-1})	0.011 (0.003)

HDL TG (mmol·L^{-1})	0.104 (0.038)
HDL VL TG (mmol·L^{-1})	0.006 (0.003)
HDL L TG (mmol·L^{-1})	0.026 (0.010)
HDL M TG (mmol·L^{-1})	0.038 (0.016)
HDL S TG (mmol·L^{-1})	0.022 (0.010)
HDL VS TG (mmol·L^{-1})	0.011 (0.003)
Total TG (mmol·L^{-1})	0.748 (0.412)
VLDL size (nm)	42.560 (3.116)
LDL size (nm)	25.766 (0.135)
HDL size (nm)	10.873 (0.212)

Abbreviations: CM = chylomicron; HDL = high-density lipoprotein; LDL = low-density lipoprotein; VLDL = very low-density lipoprotein; -C = cholesterol; -L = large; -M = medium; -PN = particle number; -S = small; -TG = triglycerides; -VL = very large; -VS = very small.

Table 2. Comparison of characteristics between those children included or not included in the analytical sample

Characteristic	Included		Excluded		Difference
	n (%)	Mean (SD)	*n* (%[a])	Mean (SD)	*p* value[b]
Baseline	762		367		
Age (years)		10.0 (0.3)	315	10.0 (0.4)	0.032
Sex					
Girls	378 (49.6)		163 (44.4)		0.102
Boys	384 (50.4)		204 (55.6)		
Anthropometry					
Height (m)		143.1 (6.7)	334	141.8 (6.9)	0.004
Weight (kg)		37.2 (8.1)	333	36.6 (7.9)	0.201
BMI ($kg \cdot m^{-2}$)		18.1 (3.0)	333	18.0 (2.9)	0.931
≥25	189 (24.8)		70 (21.0)		0.913
≥30	40 (5.2)		12 (3.6)		
Waist circumference (cm)		62.1 (7.6)	332	61.8 (7.3)	0.523
Parents' education[c]					
Upper secondary school	241 (31.6)		108 (35.2)		0.263
<4 years college/university	229 (30.1)		91 (29.6)		
≥4 years college/university	292 (38.3)		108 (35.2)		
Tanner stage[d]					
Stage 1	417 (54.7)		164 (51.4)		0.319
Stage 2	297 (39.0)		130 (40.8)		
Stage ≥3	48 (6.3)		25 (7.8)		
Physical activity					
VPA ($min \cdot d^{-1}$)		32.2 (16.2)	298	27.4 (14.9)	<0.001
MPA ($min \cdot d^{-1}$)		45.1 (12.8)	298	41.8 (14.1)	<0.001
LPA ($min \cdot d^{-1}$)		235.2 (36.7)	298	226.3 (42.5)	0.001
SED ($min \cdot d^{-1}$)		466.5 (57.5)	298	467.0 (65.2)	0.909
MVPA ≥60 ($min \cdot d^{-1}$)	563 (73.9)		189 (51.5)		<0.001
Lipid profile[e]					

TC (mmol·L^{-1})		4.5 (0.7)	246	4.4 (0.7)	0.390
LDL-C (mmol·L^{-1})		2.5 (0.6)	246	2.5 (0.7)	0.945
HDL-C (mmol·L^{-1})		1.6 (0.3)	246	1.6 (0.3)	0.116
TG (mmol·L^{-1})[f]		0.7 [0.5, 0.9]	246	0.7 [0.5, 0.9]	0.931[g]
Follow-up	720		409		
Physical activity					
VPA (min·d^{-1})		27.6 (13.4)	387	24.3 (13.3)	<0.001
MPA (min·d^{-1})		40.3 (11.9)	387	40.1 (13.2)	0.831
LPA (min·d^{-1})		220.8 (34.8)	387	218.3 (39.0)	0.276
SED (min·d^{-1})		494.3 (52.1)	387	488.6 (65.5)	0.114
MVPA ≥60 (min·d^{-1})	451 (62.6)		212 (51.8)		<0.001
Lipid profile[e]					
TC (mmol·L^{-1})		4.5 (0.6)	221	4.6 (0.7)	0.649
LDL-C (mmol·L^{-1})		2.6 (0.6)	221	2.6 (0.6)	0.641
HDL-C (mmol·L^{-1})		1.6 (0.3)	221	1.6 (0.3)	0.540
TG (mmol·L^{-1})[f]		0.6 [0.5, 0.9]	221	0.7 [0.5, 0.9]	0.224[g]

[a]Proportion of total *n* with data available.
[b]Continuous characteristics compared using linear regression, categorical characteristics using binomial logistic regression.
[c]For comparison, dichotomised to 'Upper secondary school' and 'Higher than upper secondary school'.
[d]For comparison, dichotomised to Tanner stage 1 and Tanner stage ≥2.
[e]Measured using clinical chemistry. LDL-C estimated using the Friedewald formula.
[f]Median [IQR].
[g]Comparison performed using *log*(TG).
Abbreviations: BMI = body mass index; HDL-C = high-density lipoprotein; IQR = interquartile range; LDL-C = low-density lipoprotein cholesterol; LPA = light-intensity physical activity; MPA = moderate-intensity physical activity; SD = standard deviation; SED = sedentary time; VPA = vigorous-intensity physical activity; TC = total cholesterol; TG = triglycerides.

Figure 1. Associations between change in vigorous-intensity physical activity (VPA) and follow-up lipoprotein measures

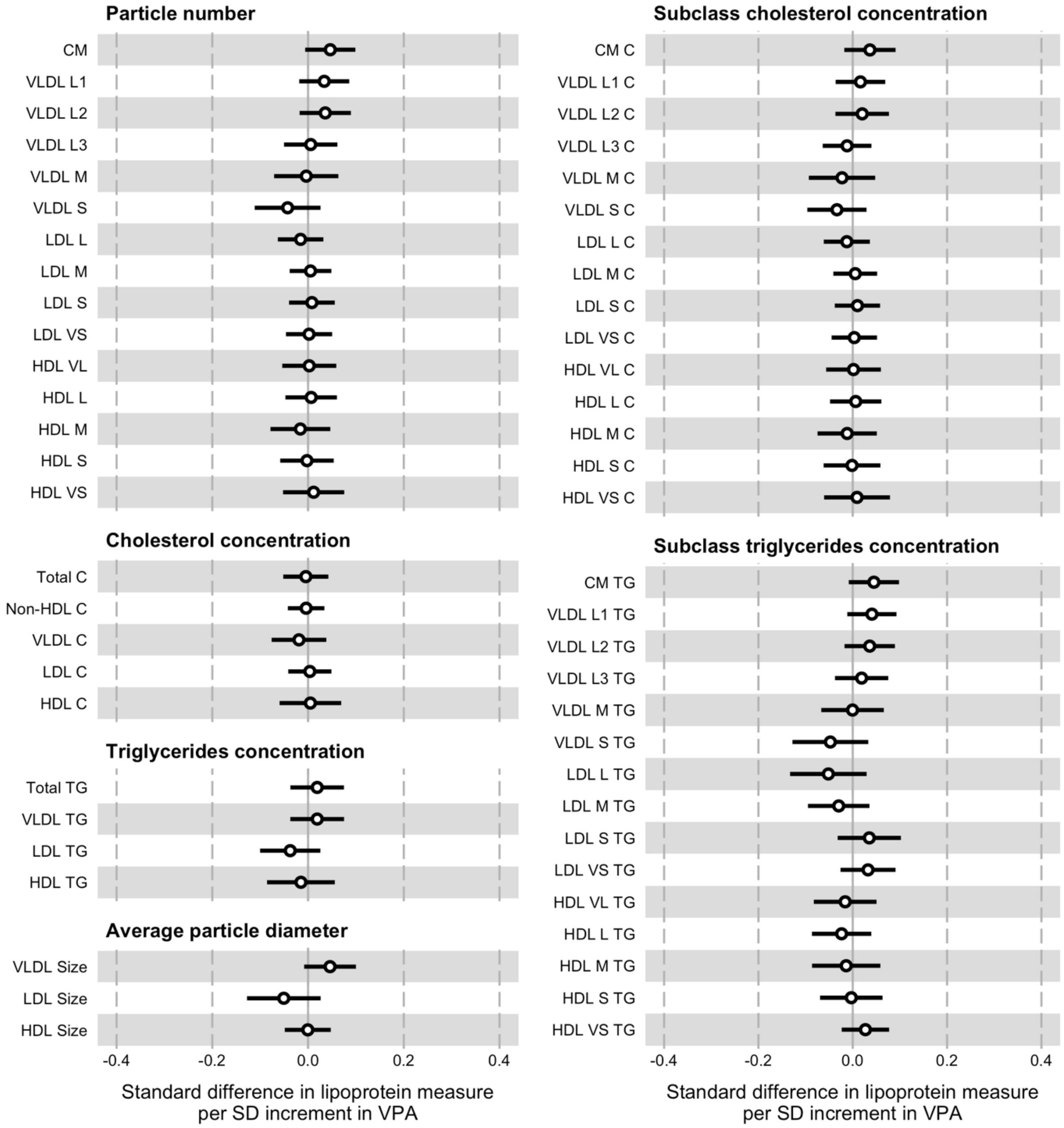

The association magnitudes are the standardised unit difference in lipoprotein measure per SD unit increment of change in activity variable (follow-up minus baseline). The models are adjusted for change in accelerometer wear time and baseline values of age, lipoprotein measure, parents' education, sex, sexual maturity, and waist circumference. Cluster-robust standard errors were calculated, clustered on the school variable. Filled circles are $p < 0.01$. Error bars are 95% confidence intervals.

Abbreviations: CM = chylomicron; HDL = high-density lipoprotein; LDL = low-density lipoprotein; SD = standard deviation; VLDL = very low-density lipoprotein; -C = cholesterol; -L = large; -M = medium; -S = small; -TG = triglycerides; -VL = very large; -VS = very small.

Figure 2. Associations between change in moderate-intensity physical activity (MPA) and follow-up lipoprotein measures

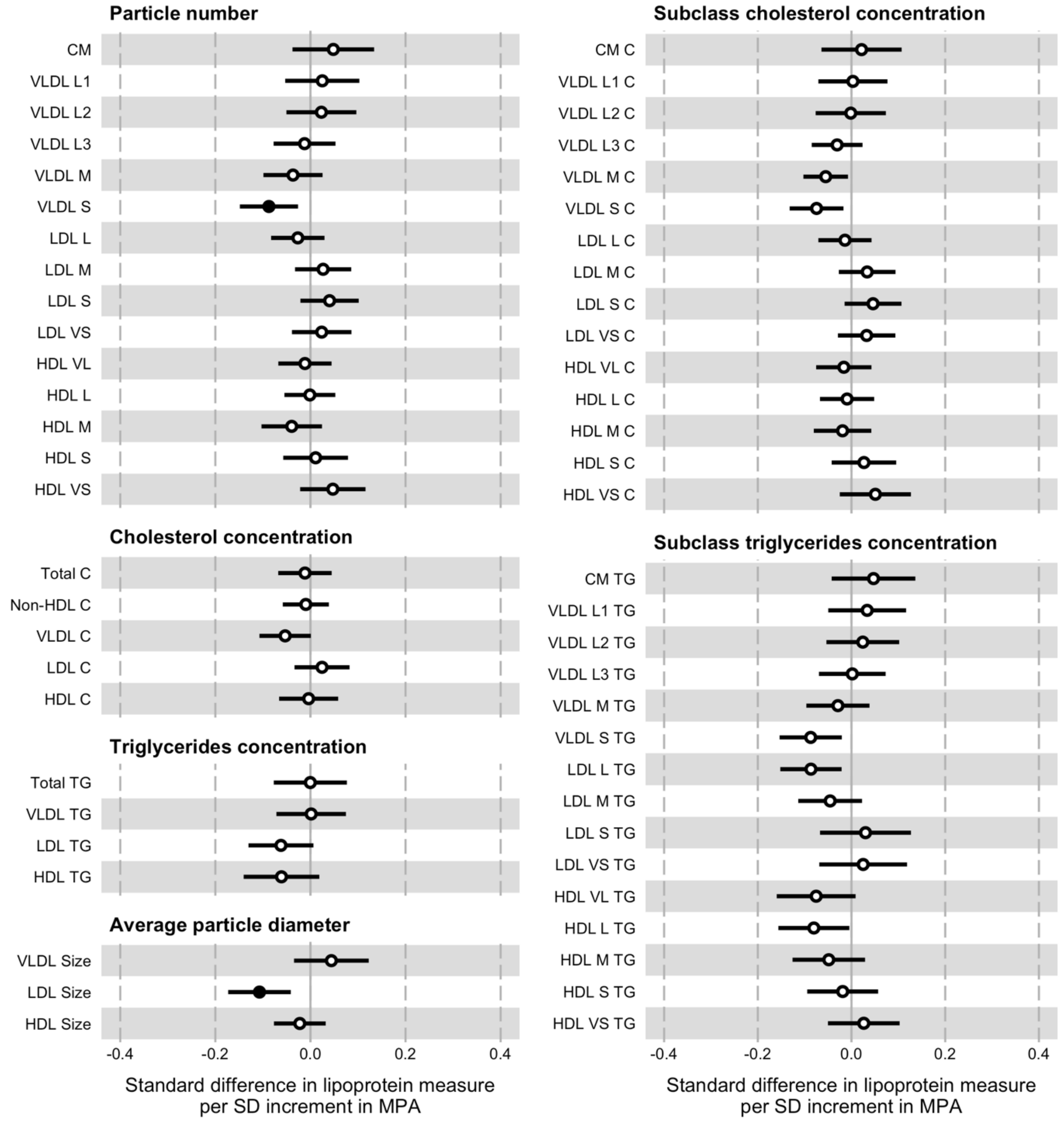

The association magnitudes are the standardised unit difference in lipoprotein measure per SD unit increment of change in activity variable (follow-up minus baseline). The models are adjusted for change in accelerometer wear time and baseline values of age, lipoprotein measure, parents' education, sex, sexual maturity, and waist circumference. Cluster-robust standard errors were calculated, clustered on the school variable. Filled circles are $p < 0.01$. Error bars are 95% confidence intervals.
Abbreviations: CM = chylomicron; HDL = high-density lipoprotein; LDL = low-density lipoprotein; SD = standard deviation; VLDL = very low-density lipoprotein; -C = cholesterol; -L = large; -M = medium; -S = small; -TG = triglycerides; -VL = very large; -VS = very small.

Figure 3. Associations between change in light-intensity physical activity (LPA) and follow-up lipoprotein measures

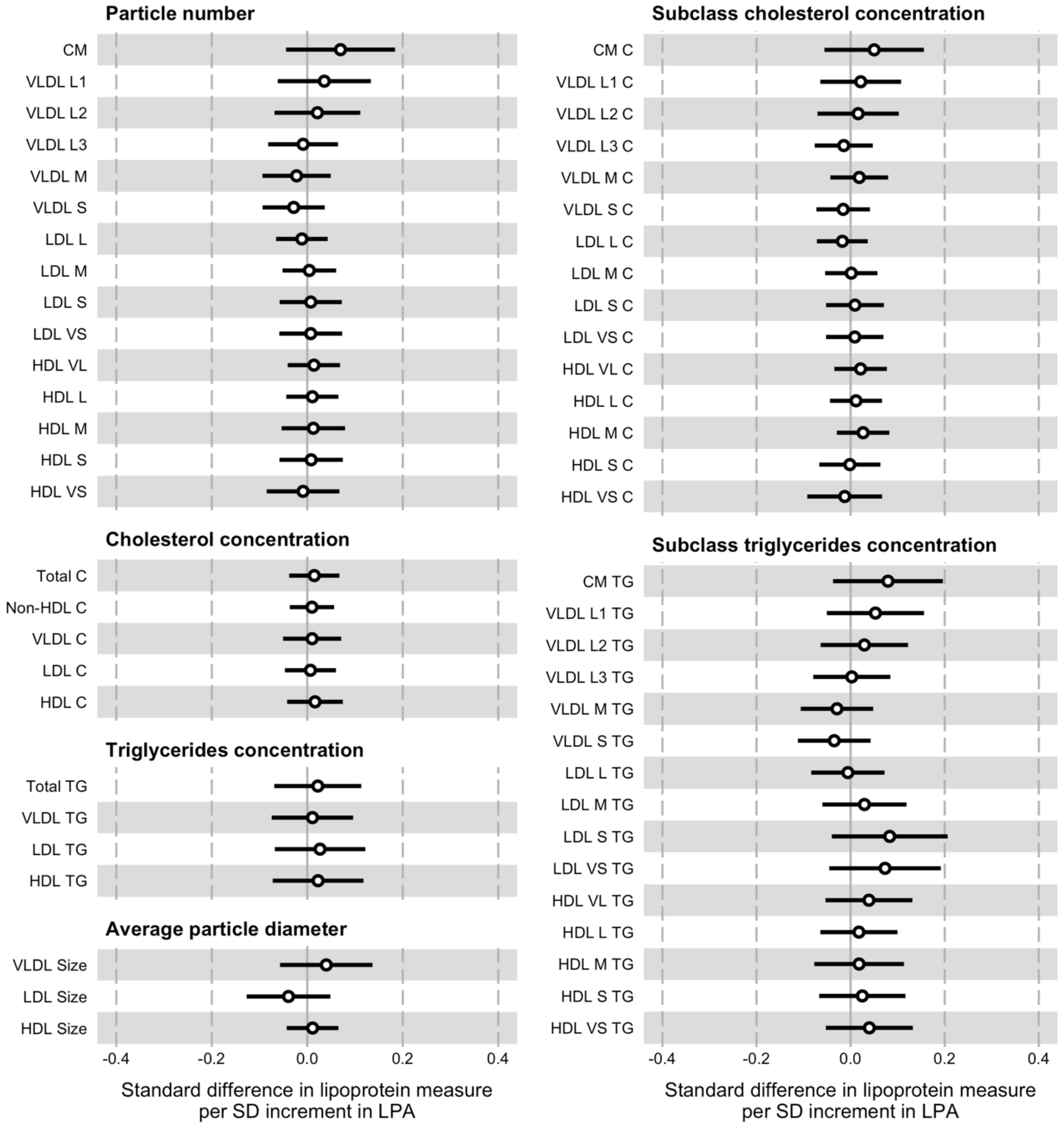

The association magnitudes are the standardised unit difference in lipoprotein measure per SD unit increment of change in activity variable (follow-up minus baseline). The models are adjusted for change in accelerometer wear time and baseline values of age, lipoprotein measure, parents' education, sex, sexual maturity, and waist circumference. Cluster-robust standard errors were calculated, clustered on the school variable. Filled circles are $p < 0.01$. Error bars are 95% confidence intervals.

Abbreviations: CM = chylomicron; HDL = high-density lipoprotein; LDL = low-density lipoprotein; SD = standard deviation; VLDL = very low-density lipoprotein; -C = cholesterol; -L = large; -M = medium; -S = small; -TG = triglycerides; -VL = very large; -VS = very small.

Figure 4. Associations between change in sedentary time and follow-up lipoprotein measures

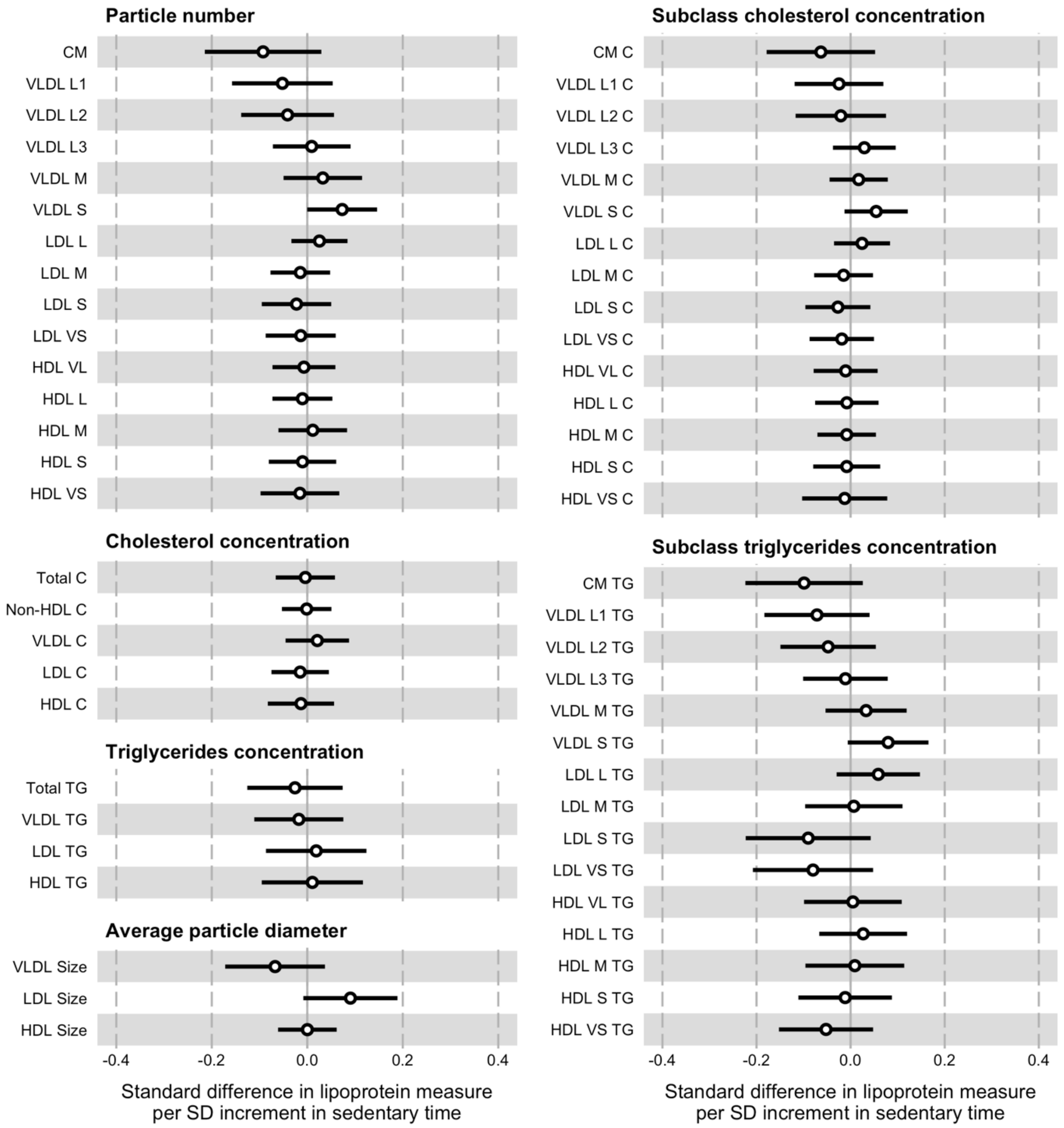

The association magnitudes are the standardised unit difference in lipoprotein measure per SD unit increment of change in activity variable (follow-up minus baseline). The models are adjusted for change in accelerometer wear time and baseline values of age, lipoprotein measure, parents' education, sex, sexual maturity, and waist circumference. Cluster-robust standard errors were calculated, clustered on the school variable. Filled circles are p <0.01. Error bars are 95% confidence intervals.

Abbreviations: CM = chylomicron; HDL = high-density lipoprotein; LDL = low-density lipoprotein; SD = standard deviation; VLDL = very low-density lipoprotein; -C = cholesterol; -L = large; -M = medium; -S = small; -TG = triglycerides; -VL = very large; -VS = very small.

Table 3. Associations between baseline vigorous-intensity physical activity (VPA) and follow-up lipoprotein measures

Lipoprotein measure	Model 1				Model 2			
	Coefficient	Lower CI	Upper CI	*p* value	Coefficient	Lower CI	Upper CI	*p* value
CM PN (nmol·L^{-1})	–0.0439	–0.0652	–0.0227	1.17E–04	–0.0304	–0.0468	–0.0140	4.71E–04
VLDL L1 PN (nmol·L^{-1})	–0.1318	–0.1983	–0.0653	2.06E–04	–0.0860	–0.1411	–0.0309	2.79E–03
VLDL L2 PN (nmol·L^{-1})	–0.4809	–0.7212	–0.2406	1.82E–04	–0.3136	–0.5226	–0.1047	3.95E–03
VLDL L3 PN (nmol·L^{-1})	–0.9019	–1.3944	–0.4095	5.44E–04	–0.5655	–1.0230	–0.1080	1.63E–02
VLDL M PN (nmol·L^{-1})	–0.8315	–1.5453	–0.1176	2.32E–02	–0.4367	–1.2215	0.3481	2.70E–01
VLDL S PN (nmol·L^{-1})	–0.1670	–0.7663	0.4322	5.79E–01	–0.0384	–0.6950	0.6183	9.07E–01
LDL L PN (nmol·L^{-1})	–1.1285	–3.0452	0.7881	2.43E–01	–0.9264	–2.9731	1.1202	3.68E–01
LDL M PN (nmol·L^{-1})	–4.5116	–9.7008	0.6776	8.71E–02	–3.8345	–9.1844	1.5155	1.57E–01
LDL S PN (nmol·L^{-1})	–2.8130	–5.8236	0.1976	6.65E–02	–2.3074	–5.2987	0.6840	1.28E–01
LDL VS PN (nmol·1^{-1})	–1.3579	–2.8924	0.1767	8.17E–02	–1.1370	–2.6677	0.3937	1.42E–01
HDL VL PN (nmol·L^{-1})	2.8404	–2.6701	8.3509	3.06E–01	1.7695	–3.6925	7.2314	5.19E–01
HDL L PN (nmol·L^{-1})	25.3609	–14.5915	65.3133	2.09E–01	17.7252	–22.0069	57.4572	3.75E–01
HDL M PN (nmol·L^{-1})	18.1486	–17.2347	53.5319	3.09E–01	8.6224	–25.6787	42.9235	6.17E–01
HDL S PN (nmol·L^{-1})	–18.4718	–50.9702	14.0266	2.60E–01	–10.4427	–42.6659	21.7806	5.19E–01
HDL VS PN (nmol·L^{-1})	–11.1428	–25.5859	3.3003	1.28E–01	–7.2357	–21.2975	6.8261	3.07E–01
CM C (mmol·L^{-1})	–0.0015	–0.0023	–0.0008	8.12E–05	–0.0010	–0.0016	–0.0005	7.39E–04
VLDL C (mmol·L^{-1})	–0.0148	–0.0243	–0.0052	3.01E–03	–0.0102	–0.0202	–0.0002	4.59E–02
VLDL L1 C (mmol·L^{-1})	–0.0018	–0.0027	–0.0009	1.23E–04	–0.0012	–0.0019	–0.0005	1.79E–03
VLDL L2 C (mmol·L^{-1})	–0.0041	–0.0062	–0.0020	2.57E–04	–0.0026	–0.0045	–0.0007	8.22E–03
VLDL L3 C (mmol·L^{-1})	–0.0058	–0.0094	–0.0021	2.58E–03	–0.0040	–0.0074	–0.0007	1.98E–02
VLDL M C (mmol·L^{-1})	–0.0048	–0.0086	–0.0011	1.27E–02	–0.0034	–0.0074	0.0005	8.63E–02
VLDL S C (mmol·L^{-1})	–0.0008	–0.0033	0.0018	5.43E–01	–0.0005	–0.0033	0.0023	7.39E–01
LDL C (mmol·L^{-1})	–0.0220	–0.0473	0.0033	8.65E–02	–0.0189	–0.0449	0.0072	1.53E–01
LDL L C (mmol·L^{-1})	–0.0030	–0.0101	0.0042	4.11E–01	–0.0024	–0.0100	0.0052	5.25E–01
LDL M C (mmol·L^{-1})	–0.0092	–0.0206	0.0023	1.16E–01	–0.0076	–0.0195	0.0042	2.01E–01
LDL S C (mmol·L^{-1})	–0.0054	–0.0109	0.0001	5.58E–02	–0.0044	–0.0099	0.0011	1.15E–01
LDL VS C (mmol·L^{-1})	–0.0020	–0.0042	0.0001	6.63E–02	–0.0017	–0.0039	0.0005	1.28E–01
HDL C (mmol·L^{-1})	0.0082	–0.0079	0.0242	3.12E–01	0.0041	–0.0116	0.0198	6.02E–01
HDL VL C (mmol·L^{-1})	0.0010	–0.0011	0.0030	3.53E–01	0.0005	–0.0015	0.0025	6.22E–01
HDL L C (mmol·L^{-1})	0.0053	–0.0032	0.0138	2.13E–01	0.0035	–0.0049	0.0119	4.07E–01
HDL M C (mmol·L^{-1})	0.0026	–0.0023	0.0076	2.90E–01	0.0011	–0.0038	0.0060	6.65E–01
HDL S C (mmol·L^{-1})	–0.0009	–0.0030	0.0013	4.34E–01	–0.0004	–0.0027	0.0018	6.94E–01

HDL VS C (mmol·L^{-1})	–0.0005	–0.0012	0.0003	2.48E–01	–0.0003	–0.0011	0.0005	4.48E–01
Total C (mmol·L^{-1})	–0.0242	–0.0567	0.0083	1.42E–01	–0.0231	–0.0566	0.0105	1.74E–01
Non-HDL C (mmol·L^{-1})	–0.0313	–0.0575	–0.0050	2.05E–02	–0.0271	–0.0528	–0.0014	3.93E–02
CM TG (mmol·L^{-1})	–0.0051	–0.0075	–0.0027	8.23E–05	–0.0036	–0.0054	–0.0018	2.30E–04
VLDL TG (mmol·L^{-1})	–0.0309	–0.0479	–0.0139	5.93E–04	–0.0193	–0.0348	–0.0037	1.60E–02
VLDL L1 TG (mmol·L^{-1})	–0.0060	–0.0089	–0.0032	8.48E–05	–0.0041	–0.0064	–0.0018	6.38E–04
VLDL L2 TG (mmol·L^{-1})	–0.0111	–0.0166	–0.0056	1.51E–04	–0.0074	–0.0120	–0.0027	2.42E–03
VLDL L3 TG (mmol·L^{-1})	–0.0110	–0.0170	–0.0050	5.56E–04	–0.0068	–0.0124	–0.0013	1.66E–02
VLDL M TG (mmol·L^{-1})	–0.0036	–0.0064	–0.0009	1.12E–02	–0.0019	–0.0049	0.0011	2.05E–01
VLDL S TG (mmol·L^{-1})	–0.0004	–0.0014	0.0006	4.46E–01	–0.0001	–0.0011	0.0010	9.11E–01
LDL TG (mmol·L^{-1})	–0.0012	–0.0031	0.0008	2.44E–01	–0.0009	–0.0028	0.0011	3.78E–01
LDL L TG (mmol·L^{-1})	–0.0003	–0.0016	0.0009	5.95E–01	–0.0001	–0.0014	0.0011	8.26E–01
LDL M TG (mmol·L^{-1})	–0.0003	–0.0012	0.0005	4.26E–01	–0.0003	–0.0011	0.0005	4.83E–01
LDL S TG (mmol·L^{-1})	–0.0007	–0.0010	–0.0003	4.40E–04	–0.0005	–0.0008	–0.0002	8.12E–04
LDL VS TG (mmol·L^{-1})	–0.0004	–0.0006	–0.0002	4.51E–05	–0.0003	–0.0004	–0.0002	4.20E–05
HDL TG (mmol·L^{-1})	–0.0014	–0.0037	0.0009	2.33E–01	–0.0008	–0.0032	0.0017	5.43E–01
HDL VL TG (mmol·L^{-1})	0.0000	–0.0001	0.0001	8.32E–01	0.0000	–0.0002	0.0001	5.00E–01
HDL L TG (mmol·L^{-1})	0.0004	–0.0003	0.0010	2.40E–01	0.0002	–0.0004	0.0009	5.24E–01
HDL M TG (mmol·L^{-1})	–0.0007	–0.0018	0.0004	1.93E–01	–0.0003	–0.0015	0.0008	5.82E–01
HDL S TG (mmol·L^{-1})	–0.0007	–0.0014	0.0000	4.77E–02	–0.0004	–0.0011	0.0003	3.04E–01
HDL VS TG (mmol·L^{-1})	–0.0003	–0.0005	–0.0002	1.14E–04	–0.0002	–0.0004	–0.0001	1.24E–03
Total TG (mmol·L^{-1})	–0.0399	–0.0613	–0.0186	4.34E–04	–0.0254	–0.0447	–0.0061	1.07E–02
VLDL size (nm)	–0.3647	–0.5437	–0.1856	1.44E–04	–0.2539	–0.4038	–0.1041	1.27E–03
LDL size (nm)	0.0104	–0.0007	0.0215	6.59E–02	0.0084	–0.0028	0.0196	1.38E–01
HDL size (nm)	0.0077	–0.0029	0.0183	1.52E–01	0.0051	–0.0056	0.0157	3.45E–01

Regression coefficients are in absolute concentration units of lipoprotein measures per SD unit increment of activity variable.
Model 1 is adjusted for baseline values of accelerometer wear time, age, lipoprotein measure, parents' education, sex, and sexual maturity. Cluster-robust standard errors were calculated, clustered on the school variable.
Model 2 is adjusted for baseline waist circumference in addition to the Model 1 covariates.
p values should be interpreted at a Bonferroni-corrected threshold of 0.01.
In notation of *p* values 1.23E–02 stands for "1.23 times 10 to the power of –02" or 0.0123.
Abbreviations: CI = confidence interval; CM = chylomicron; HDL = high-density lipoprotein; LDL = low-density lipoprotein; SD = standard deviation; VLDL = very low-density lipoprotein; -C = cholesterol; -L = large; -M = medium; -PN = particle number; -S = small; -TG = triglycerides; -VL = very large; -VS = very small.

Table 4. Associations between baseline moderate-intensity physical activity (MPA) and follow-up lipoprotein measures

Lipoprotein measure	Model 1				Model 2			
	Coefficient	Lower CI	Upper CI	*p* value	Coefficient	Lower CI	Upper CI	*p* value
CM PN (nmol·L^{-1})	–0.0382	–0.0642	–0.0122	4.78E–03	–0.0301	–0.0548	–0.0055	1.75E–02
VLDL L1 PN (nmol·L^{-1})	–0.1139	–0.1973	–0.0306	8.29E–03	–0.0876	–0.1657	–0.0096	2.84E–02
VLDL L2 PN (nmol·L^{-1})	–0.4173	–0.7279	–0.1067	9.37E–03	–0.3211	–0.6129	–0.0294	3.16E–02
VLDL L3 PN (nmol·L^{-1})	–0.7346	–1.4111	–0.0581	3.38E–02	–0.5477	–1.1816	0.0862	8.90E–02
VLDL M PN (nmol·L^{-1})	–0.5595	–1.3445	0.2255	1.59E–01	–0.3334	–1.0787	0.4120	3.74E–01
VLDL S PN (nmol·L^{-1})	0.1848	–0.4951	0.8647	5.88E–01	0.2700	–0.4295	0.9695	4.43E–01
LDL L PN (nmol·L^{-1})	0.3586	–2.3233	3.0405	7.90E–01	0.5238	–2.2372	3.2847	7.05E–01
LDL M PN (nmol·L^{-1})	–1.9253	–8.2246	4.3741	5.43E–01	–1.4106	–7.9024	5.0812	6.65E–01
LDL S PN (nmol·L^{-1})	–2.0133	–5.3129	1.2863	2.27E–01	–1.6625	–5.0433	1.7183	3.29E–01
LDL VS PN (nmol·1^{-1})	–1.0403	–2.6494	0.5689	2.01E–01	–0.8857	–2.5288	0.7574	2.85E–01
HDL VL PN (nmol·L^{-1})	–1.0764	–6.0858	3.9331	6.69E–01	–1.7402	–6.8905	3.4102	5.01E–01
HDL L PN (nmol·L^{-1})	–6.4509	–46.3380	33.4362	7.47E–01	–10.9988	–52.0761	30.0785	5.94E–01
HDL M PN (nmol·L^{-1})	9.9821	–34.2439	54.2081	6.53E–01	4.3478	–40.5196	49.2152	8.47E–01
HDL S PN (nmol·L^{-1})	–1.0307	–42.7645	40.7031	9.61E–01	4.5043	–38.3276	47.3362	8.34E–01
HDL VS PN (nmol·L^{-1})	–8.3757	–28.0431	11.2917	3.97E–01	–5.7361	–26.3380	14.8658	5.79E–01
CM C (mmol·L^{-1})	–0.0014	–0.0023	–0.0005	4.35E–03	–0.0011	–0.0020	–0.0002	1.50E–02
VLDL C (mmol·L^{-1})	–0.0078	–0.0193	0.0037	1.81E–01	–0.0052	–0.0162	0.0059	3.55E–01
VLDL L1 C (mmol·L^{-1})	–0.0015	–0.0026	–0.0004	9.61E–03	–0.0012	–0.0022	–0.0001	2.90E–02
VLDL L2 C (mmol·L^{-1})	–0.0035	–0.0062	–0.0008	1.26E–02	–0.0026	–0.0052	–0.0001	3.98E–02
VLDL L3 C (mmol·L^{-1})	–0.0047	–0.0098	0.0003	6.53E–02	–0.0037	–0.0086	0.0011	1.25E–01
VLDL M C (mmol·L^{-1})	–0.0023	–0.0056	0.0010	1.65E–01	–0.0015	–0.0047	0.0017	3.53E–01
VLDL S C (mmol·L^{-1})	0.0008	–0.0022	0.0038	5.90E–01	0.0010	–0.0021	0.0042	5.12E–01
LDL C (mmol·L^{-1})	–0.0120	–0.0427	0.0187	4.38E–01	–0.0096	–0.0412	0.0220	5.46E–01
LDL L C (mmol·L^{-1})	0.0007	–0.0087	0.0100	8.90E–01	0.0011	–0.0087	0.0108	8.24E–01
LDL M C (mmol·L^{-1})	–0.0049	–0.0191	0.0094	4.94E–01	–0.0038	–0.0185	0.0109	6.08E–01
LDL S C (mmol·L^{-1})	–0.0040	–0.0101	0.0022	2.00E–01	–0.0033	–0.0096	0.0030	3.00E–01
LDL VS C (mmol·L^{-1})	–0.0015	–0.0039	0.0008	1.87E–01	–0.0013	–0.0037	0.0011	2.76E–01
HDL C (mmol·L^{-1})	–0.0012	–0.0177	0.0152	8.81E–01	–0.0034	–0.0199	0.0130	6.78E–01
HDL VL C (mmol·L^{-1})	–0.0006	–0.0025	0.0014	5.63E–01	–0.0008	–0.0028	0.0011	4.02E–01
HDL L C (mmol·L^{-1})	–0.0013	–0.0098	0.0071	7.56E–01	–0.0024	–0.0111	0.0063	5.86E–01
HDL M C (mmol·L^{-1})	0.0005	–0.0055	0.0065	8.72E–01	–0.0004	–0.0064	0.0056	8.92E–01
HDL S C (mmol·L^{-1})	0.0002	–0.0027	0.0031	8.84E–01	0.0005	–0.0025	0.0035	7.41E–01

HDL VS C (mmol·L^{-1})	–0.0003	–0.0014	0.0007	5.17E–01	–0.0002	–0.0013	0.0008	6.70E–01
Total C (mmol·L^{-1})	–0.0158	–0.0577	0.0260	4.51E–01	–0.0147	–0.0572	0.0277	4.90E–01
Non-HDL C (mmol·L^{-1})	–0.0140	–0.0498	0.0217	4.35E–01	–0.0110	–0.0464	0.0245	5.38E–01
CM TG (mmol·L^{-1})	–0.0045	–0.0074	–0.0015	3.57E–03	–0.0036	–0.0064	–0.0008	1.34E–02
VLDL TG (mmol·L^{-1})	–0.0258	–0.0477	–0.0040	2.15E–02	–0.0192	–0.0397	0.0014	6.70E–02
VLDL L1 TG (mmol·L^{-1})	–0.0053	–0.0089	–0.0017	4.61E–03	–0.0042	–0.0075	–0.0008	1.68E–02
VLDL L2 TG (mmol·L^{-1})	–0.0096	–0.0167	–0.0025	8.82E–03	–0.0074	–0.0141	–0.0007	3.00E–02
VLDL L3 TG (mmol·L^{-1})	–0.0092	–0.0171	–0.0012	2.41E–02	–0.0068	–0.0142	0.0006	7.25E–02
VLDL M TG (mmol·L^{-1})	–0.0027	–0.0061	0.0006	1.07E–01	–0.0017	–0.0049	0.0015	2.81E–01
VLDL S TG (mmol·L^{-1})	–0.0001	–0.0010	0.0007	7.50E–01	0.0001	–0.0008	0.0009	8.94E–01
LDL TG (mmol·L^{-1})	–0.0005	–0.0027	0.0016	6.26E–01	–0.0003	–0.0025	0.0018	7.58E–01
LDL L TG (mmol·L^{-1})	0.0000	–0.0012	0.0012	9.75E–01	0.0001	–0.0010	0.0013	8.02E–01
LDL M TG (mmol·L^{-1})	–0.0001	–0.0011	0.0009	8.76E–01	0.0000	–0.0011	0.0010	9.32E–01
LDL S TG (mmol·L^{-1})	–0.0007	–0.0011	–0.0003	1.32E–03	–0.0006	–0.0010	–0.0002	3.01E–03
LDL VS TG (mmol·L^{-1})	–0.0004	–0.0006	–0.0001	1.56E–03	–0.0003	–0.0005	–0.0001	4.76E–03
HDL TG (mmol·L^{-1})	–0.0013	–0.0035	0.0010	2.73E–01	–0.0009	–0.0032	0.0014	4.59E–01
HDL VL TG (mmol·L^{-1})	–0.0001	–0.0002	0.0001	3.22E–01	–0.0001	–0.0003	0.0001	2.29E–01
HDL L TG (mmol·L^{-1})	0.0002	–0.0005	0.0008	6.25E–01	0.0000	–0.0006	0.0007	9.04E–01
HDL M TG (mmol·L^{-1})	–0.0004	–0.0014	0.0005	3.67E–01	–0.0002	–0.0012	0.0008	6.82E–01
HDL S TG (mmol·L^{-1})	–0.0004	–0.0011	0.0002	1.74E–01	–0.0002	–0.0009	0.0004	4.39E–01
HDL VS TG (mmol·L^{-1})	–0.0003	–0.0005	–0.0001	8.11E–03	–0.0002	–0.0004	0.0000	2.94E–02
Total TG (mmol·L^{-1})	–0.0337	–0.0610	–0.0064	1.66E–02	–0.0253	–0.0510	0.0005	5.42E–02
VLDL size (nm)	–0.3250	–0.5441	–0.1059	4.36E–03	–0.2601	–0.4685	–0.0516	1.54E–02
LDL size (nm)	0.0121	0.0016	0.0226	2.46E–02	0.0109	–0.0002	0.0219	5.40E–02
HDL size (nm)	–0.0019	–0.0131	0.0093	7.35E–01	–0.0035	–0.0152	0.0082	5.50E–01

Regression coefficients are in absolute concentration units of lipoprotein measures per SD unit increment of activity variable.
Model 1 is adjusted for baseline values of accelerometer wear time, age, lipoprotein measure, parents' education, sex, and sexual maturity. Cluster-robust standard errors were calculated, clustered on the school variable.
Model 2 is adjusted for baseline waist circumference in addition to the Model 1 covariates.
p values should be interpreted at a Bonferroni-corrected threshold of 0.01.
In notation of *p* values 1.23E–02 stands for "1.23 times 10 to the power of –02" or 0.0123.
Abbreviations: CI = confidence interval; CM = chylomicron; HDL = high-density lipoprotein; LDL = low-density lipoprotein; SD = standard deviation; VLDL = very low-density lipoprotein; -C = cholesterol; -L = large; -M = medium; -PN = particle number; -S = small; -TG = triglycerides; -VL = very large; -VS = very small.

Table 5. Associations between baseline light-intensity physical activity (LPA) and follow-up lipoprotein measures

Lipoprotein measure	Model 1				Model 2			
	Coefficient	Lower CI	Upper CI	*p* value	Coefficient	Lower CI	Upper CI	*p* value
CM PN (nmol·L^{-1})	–0.0185	–0.0431	0.0062	1.40E–01	–0.0172	–0.0415	0.0070	1.60E–01
VLDL L1 PN (nmol·L^{-1})	–0.0453	–0.1214	0.0309	2.39E–01	–0.0419	–0.1143	0.0305	2.51E–01
VLDL L2 PN (nmol·L^{-1})	–0.1762	–0.4437	0.0914	1.93E–01	–0.1652	–0.4170	0.0866	1.94E–01
VLDL L3 PN (nmol·L^{-1})	–0.0125	–0.5968	0.5717	9.66E–01	0.0079	–0.5278	0.5436	9.76E–01
VLDL M PN (nmol·L^{-1})	0.0810	–0.6827	0.8446	8.33E–01	0.1006	–0.6010	0.8022	7.75E–01
VLDL S PN (nmol·L^{-1})	0.5186	–0.3181	1.3553	2.20E–01	0.5320	–0.3045	1.3686	2.08E–01
LDL L PN (nmol·L^{-1})	2.5262	0.0932	4.9591	4.21E–02	2.5597	0.1172	5.0022	4.03E–02
LDL M PN (nmol·L^{-1})	5.4295	0.1334	10.7256	4.47E–02	5.5798	0.3067	10.8529	3.85E–02
LDL S PN (nmol·L^{-1})	2.4162	–0.5966	5.4289	1.14E–01	2.5299	–0.4694	5.5293	9.66E–02
LDL VS PN (nmol·1^{-1})	0.7679	–0.6816	2.2175	2.93E–01	0.8123	–0.6372	2.2618	2.66E–01
HDL VL PN (nmol·L^{-1})	–7.5808	–12.0786	–3.0830	1.34E–03	–7.7217	–12.0542	–3.3893	7.41E–04
HDL L PN (nmol·L^{-1})	–50.7116	–83.0841	–18.3390	2.71E–03	–51.6776	–82.9954	–20.3598	1.66E–03
HDL M PN (nmol·L^{-1})	–22.6285	–58.4923	13.2353	2.11E–01	–23.6760	–58.9766	11.6246	1.85E–01
HDL S PN (nmol·L^{-1})	34.9046	–3.9806	73.7898	7.75E–02	36.1746	–2.2388	74.5881	6.44E–02
HDL VS PN (nmol·L^{-1})	16.0605	–1.1641	33.2851	6.70E–02	16.7175	–0.4156	33.8506	5.56E–02
CM C (mmol·L^{-1})	–0.0007	–0.0015	0.0002	1.44E–01	–0.0006	–0.0015	0.0002	1.55E–01
VLDL C (mmol·L^{-1})	0.0016	–0.0118	0.0150	8.10E–01	0.0020	–0.0109	0.0149	7.59E–01
VLDL L1 C (mmol·L^{-1})	–0.0003	–0.0013	0.0007	5.59E–01	–0.0003	–0.0012	0.0007	5.85E–01
VLDL L2 C (mmol·L^{-1})	–0.0010	–0.0034	0.0014	4.06E–01	–0.0009	–0.0032	0.0013	4.13E–01
VLDL L3 C (mmol·L^{-1})	0.0018	–0.0023	0.0059	3.92E–01	0.0019	–0.0020	0.0058	3.29E–01
VLDL M C (mmol·L^{-1})	–0.0011	–0.0049	0.0028	5.84E–01	–0.0010	–0.0047	0.0027	5.91E–01
VLDL S C (mmol·L^{-1})	0.0017	–0.0021	0.0054	3.72E–01	0.0017	–0.0021	0.0055	3.66E–01
LDL C (mmol·L^{-1})	0.0212	–0.0048	0.0471	1.08E–01	0.0218	–0.0041	0.0477	9.69E–02
LDL L C (mmol·L^{-1})	0.0085	0.0002	0.0168	4.58E–02	0.0086	0.0002	0.0170	4.55E–02
LDL M C (mmol·L^{-1})	0.0127	0.0009	0.0246	3.52E–02	0.0131	0.0013	0.0248	3.05E–02
LDL S C (mmol·L^{-1})	0.0041	–0.0015	0.0096	1.45E–01	0.0043	–0.0012	0.0098	1.24E–01
LDL VS C (mmol·L^{-1})	0.0012	–0.0009	0.0033	2.52E–01	0.0013	–0.0008	0.0034	2.27E–01
HDL C (mmol·L^{-1})	–0.0152	–0.0277	–0.0027	1.79E–02	–0.0155	–0.0274	–0.0036	1.19E–02
HDL VL C (mmol·L^{-1})	–0.0027	–0.0045	–0.0009	3.19E–03	–0.0028	–0.0045	–0.0011	1.85E–03
HDL L C (mmol·L^{-1})	–0.0109	–0.0185	–0.0034	5.22E–03	–0.0112	–0.0184	–0.0039	3.30E–03
HDL M C (mmol·L^{-1})	–0.0037	–0.0081	0.0008	1.04E–01	–0.0038	–0.0080	0.0004	7.61E–02
HDL S C (mmol·L^{-1})	0.0024	–0.0002	0.0051	7.32E–02	0.0025	–0.0002	0.0051	6.51E–02

HDL VS C (mmol·L^{-1})	0.0007	–0.0002	0.0016	1.06E–01	0.0008	–0.0001	0.0017	9.79E–02
Total C (mmol·L^{-1})	0.0096	–0.0258	0.0449	5.90E–01	0.0098	–0.0255	0.0452	5.79E–01
Non-HDL C (mmol·L^{-1})	0.0233	–0.0097	0.0562	1.63E–01	0.0241	–0.0085	0.0568	1.45E–01
CM TG (mmol·L^{-1})	–0.0020	–0.0048	0.0008	1.57E–01	–0.0019	–0.0046	0.0009	1.82E–01
VLDL TG (mmol·L^{-1})	–0.0052	–0.0243	0.0138	5.85E–01	–0.0045	–0.0223	0.0133	6.14E–01
VLDL L1 TG (mmol·L^{-1})	–0.0022	–0.0054	0.0010	1.69E–01	–0.0021	–0.0052	0.0010	1.84E–01
VLDL L2 TG (mmol·L^{-1})	–0.0034	–0.0095	0.0027	2.68E–01	–0.0031	–0.0089	0.0026	2.82E–01
VLDL L3 TG (mmol·L^{-1})	–0.0014	–0.0081	0.0053	6.85E–01	–0.0011	–0.0073	0.0051	7.20E–01
VLDL M TG (mmol·L^{-1})	0.0006	–0.0026	0.0037	7.23E–01	0.0007	–0.0023	0.0036	6.56E–01
VLDL S TG (mmol·L^{-1})	0.0007	–0.0004	0.0017	2.02E–01	0.0007	–0.0003	0.0017	1.77E–01
LDL TG (mmol·L^{-1})	0.0010	–0.0015	0.0035	4.36E–01	0.0010	–0.0015	0.0035	4.26E–01
LDL L TG (mmol·L^{-1})	0.0007	–0.0005	0.0019	2.65E–01	0.0007	–0.0005	0.0020	2.53E–01
LDL M TG (mmol·L^{-1})	0.0003	–0.0007	0.0014	5.48E–01	0.0003	–0.0007	0.0014	5.42E–01
LDL S TG (mmol·L^{-1})	–0.0004	–0.0008	0.0000	5.07E–02	–0.0004	–0.0008	0.0000	5.89E–02
LDL VS TG (mmol·L^{-1})	–0.0002	–0.0004	0.0000	8.27E–02	–0.0002	–0.0004	0.0000	9.61E–02
HDL TG (mmol·L^{-1})	–0.0009	–0.0036	0.0018	5.09E–01	–0.0009	–0.0036	0.0019	5.30E–01
HDL VL TG (mmol·L^{-1})	–0.0002	–0.0004	0.0000	1.85E–02	–0.0002	–0.0004	0.0000	1.61E–02
HDL L TG (mmol·L^{-1})	–0.0003	–0.0010	0.0004	3.80E–01	–0.0003	–0.0010	0.0003	3.41E–01
HDL M TG (mmol·L^{-1})	0.0000	–0.0012	0.0012	9.99E–01	0.0000	–0.0011	0.0012	9.61E–01
HDL S TG (mmol·L^{-1})	0.0002	–0.0005	0.0009	6.41E–01	0.0002	–0.0005	0.0009	5.77E–01
HDL VS TG (mmol·L^{-1})	0.0000	–0.0002	0.0001	6.11E–01	0.0000	–0.0002	0.0001	6.56E–01
Total TG (mmol·L^{-1})	–0.0093	–0.0342	0.0156	4.57E–01	–0.0083	–0.0319	0.0152	4.81E–01
VLDL size (nm)	–0.1168	–0.2955	0.0620	1.96E–01	–0.1087	–0.2802	0.0627	2.09E–01
LDL size (nm)	0.0015	–0.0100	0.0130	7.94E–01	0.0013	–0.0103	0.0129	8.25E–01
HDL size (nm)	–0.0158	–0.0261	–0.0055	3.34E–03	–0.0162	–0.0261	–0.0064	1.72E–03

Regression coefficients are in absolute concentration units of lipoprotein measures per SD unit increment of activity variable.
Model 1 is adjusted for baseline values of accelerometer wear time, age, lipoprotein measure, parents' education, sex, and sexual maturity. Cluster-robust standard errors were calculated, clustered on the school variable.
Model 2 is adjusted for baseline waist circumference in addition to the Model 1 covariates.
p values should be interpreted at a Bonferroni-corrected threshold of 0.01.
In notation of *p* values 1.23E–02 stands for "1.23 times 10 to the power of –02" or 0.0123.
Abbreviations: CI = confidence interval; CM = chylomicron; HDL = high-density lipoprotein; LDL = low-density lipoprotein; SD = standard deviation; VLDL = very low-density lipoprotein; -C = cholesterol; -L = large; -M = medium; -PN = particle number; -S = small; -TG = triglycerides; -VL = very large; -VS = very small.

Table 6. Associations between baseline sedentary time and follow-up lipoprotein measures

Lipoprotein measure	Model 1				Model 2			
	Coefficient	Lower CI	Upper CI	*p* value	Coefficient	Lower CI	Upper CI	*p* value
CM PN (nmol·L^{-1})	0.0435	0.0141	0.0728	4.45E–03	0.0346	0.0074	0.0617	1.35E–02
VLDL L1 PN (nmol·L^{-1})	0.1218	0.0304	0.2132	9.92E–03	0.0926	0.0097	0.1754	2.92E–02
VLDL L2 PN (nmol·L^{-1})	0.4531	0.1276	0.7787	7.23E–03	0.3476	0.0522	0.6430	2.19E–02
VLDL L3 PN (nmol·L^{-1})	0.5786	–0.1062	1.2634	9.61E–02	0.3684	–0.2470	0.9838	2.36E–01
VLDL M PN (nmol·L^{-1})	0.4283	–0.4507	1.3074	3.33E–01	0.1824	–0.6441	1.0088	6.60E–01
VLDL S PN (nmol·L^{-1})	–0.3936	–1.2882	0.5011	3.82E–01	–0.4876	–1.4312	0.4560	3.05E–01
LDL L PN (nmol·L^{-1})	–1.6423	–4.7158	1.4313	2.89E–01	–1.8430	–5.0281	1.3421	2.51E–01
LDL M PN (nmol·L^{-1})	–1.9206	–8.9266	5.0854	5.85E–01	–2.5777	–9.6757	4.5202	4.70E–01
LDL S PN (nmol·L^{-1})	–0.2138	–4.0090	3.5815	9.11E–01	–0.6610	–4.4907	3.1687	7.31E–01
LDL VS PN (nmol·1^{-1})	0.2335	–1.6192	2.0863	8.02E–01	0.0429	–1.8255	1.9114	9.63E–01
HDL VL PN (nmol·L^{-1})	5.2069	0.0091	10.4048	4.96E–02	6.0354	0.8816	11.1893	2.25E–02
HDL L PN (nmol·L^{-1})	32.2079	–6.2829	70.6988	9.93E–02	37.9489	–0.1736	76.0714	5.10E–02
HDL M PN (nmol·L^{-1})	8.5382	–35.2908	52.3673	6.98E–01	15.1323	–28.6523	58.9169	4.92E–01
HDL S PN (nmol·L^{-1})	–19.3194	–60.5987	21.9599	3.53E–01	–25.7449	–66.8657	15.3760	2.15E–01
HDL VS PN (nmol·L^{-1})	–5.6793	–25.8369	14.4783	5.75E–01	–8.8155	–29.5220	11.8910	3.97E–01
CM C (mmol·L^{-1})	0.0015	0.0005	0.0026	5.03E–03	0.0012	0.0003	0.0022	1.44E–02
VLDL C (mmol·L^{-1})	0.0068	–0.0076	0.0213	3.48E–01	0.0038	–0.0102	0.0178	5.87E–01
VLDL L1 C (mmol·L^{-1})	0.0014	0.0002	0.0026	2.46E–02	0.0010	–0.0001	0.0021	6.35E–02
VLDL L2 C (mmol·L^{-1})	0.0034	0.0005	0.0063	2.12E–02	0.0025	–0.0001	0.0051	5.91E–02
VLDL L3 C (mmol·L^{-1})	0.0022	–0.0029	0.0073	3.89E–01	0.0011	–0.0037	0.0058	6.56E–01
VLDL M C (mmol·L^{-1})	0.0034	–0.0007	0.0076	1.06E–01	0.0026	–0.0015	0.0067	2.13E–01
VLDL S C (mmol·L^{-1})	–0.0012	–0.0052	0.0027	5.35E–01	–0.0015	–0.0058	0.0028	4.90E–01
LDL C (mmol·L^{-1})	–0.0044	–0.0389	0.0300	7.98E–01	–0.0074	–0.0423	0.0275	6.72E–01
LDL L C (mmol·L^{-1})	–0.0057	–0.0160	0.0046	2.72E–01	–0.0062	–0.0170	0.0045	2.51E–01
LDL M C (mmol·L^{-1})	–0.0050	–0.0206	0.0107	5.28E–01	–0.0064	–0.0223	0.0095	4.24E–01
LDL S C (mmol·L^{-1})	0.0000	–0.0070	0.0071	9.89E–01	–0.0008	–0.0079	0.0063	8.22E–01
LDL VS C (mmol·L^{-1})	0.0003	–0.0024	0.0030	8.30E–01	0.0000	–0.0027	0.0027	9.99E–01
HDL C (mmol·L^{-1})	0.0094	–0.0066	0.0254	2.46E–01	0.0120	–0.0034	0.0275	1.25E–01
HDL VL C (mmol·L^{-1})	0.0019	–0.0001	0.0040	6.11E–02	0.0023	0.0003	0.0043	2.75E–02
HDL L C (mmol·L^{-1})	0.0070	–0.0017	0.0156	1.11E–01	0.0083	–0.0003	0.0169	5.71E–02
HDL M C (mmol·L^{-1})	0.0018	–0.0039	0.0075	5.28E–01	0.0029	–0.0027	0.0084	3.02E–01
HDL S C (mmol·L^{-1})	–0.0016	–0.0046	0.0014	2.82E–01	–0.0019	–0.0049	0.0010	1.98E–01

HDL VS C (mmol·L^{-1})	–0.0003	–0.0014	0.0008	5.95E–01	–0.0004	–0.0016	0.0007	4.71E–01
Total C (mmol·L^{-1})	0.0068	–0.0405	0.0542	7.74E–01	0.0054	–0.0425	0.0533	8.22E–01
Non-HDL C (mmol·L^{-1})	–0.0019	–0.0440	0.0402	9.28E–01	–0.0057	–0.0474	0.0359	7.84E–01
CM TG (mmol·L^{-1})	0.0049	0.0016	0.0083	4.21E–03	0.0039	0.0009	0.0070	1.29E–02
VLDL TG (mmol·L^{-1})	0.0240	0.0015	0.0464	3.70E–02	0.0166	–0.0038	0.0371	1.08E–01
VLDL L1 TG (mmol·L^{-1})	0.0057	0.0018	0.0096	4.81E–03	0.0045	0.0009	0.0080	1.46E–02
VLDL L2 TG (mmol·L^{-1})	0.0099	0.0025	0.0173	9.50E–03	0.0075	0.0008	0.0142	2.90E–02
VLDL L3 TG (mmol·L^{-1})	0.0081	0.0002	0.0160	4.51E–02	0.0055	–0.0017	0.0127	1.29E–01
VLDL M TG (mmol·L^{-1})	0.0018	–0.0017	0.0053	3.02E–01	0.0007	–0.0025	0.0040	6.60E–01
VLDL S TG (mmol·L^{-1})	–0.0003	–0.0014	0.0008	5.55E–01	–0.0005	–0.0017	0.0006	3.34E–01
LDL TG (mmol·L^{-1})	–0.0001	–0.0031	0.0028	9.24E–01	–0.0004	–0.0032	0.0025	8.08E–01
LDL L TG (mmol·L^{-1})	–0.0004	–0.0019	0.0011	5.87E–01	–0.0005	–0.0020	0.0009	4.65E–01
LDL M TG (mmol·L^{-1})	–0.0001	–0.0013	0.0012	8.95E–01	–0.0001	–0.0014	0.0011	8.46E–01
LDL S TG (mmol·L^{-1})	0.0008	0.0003	0.0012	1.45E–03	0.0007	0.0002	0.0011	2.75E–03
LDL VS TG (mmol·L^{-1})	0.0004	0.0002	0.0007	1.69E–03	0.0003	0.0001	0.0006	4.14E–03
HDL TG (mmol·L^{-1})	0.0017	–0.0012	0.0046	2.54E–01	0.0013	–0.0017	0.0043	4.02E–01
HDL VL TG (mmol·L^{-1})	0.0002	0.0000	0.0004	4.71E–02	0.0002	0.0000	0.0004	2.76E–02
HDL L TG (mmol·L^{-1})	0.0000	–0.0007	0.0008	9.25E–01	0.0002	–0.0006	0.0009	6.65E–01
HDL M TG (mmol·L^{-1})	0.0004	–0.0008	0.0017	4.96E–01	0.0002	–0.0011	0.0014	7.86E–01
HDL S TG (mmol·L^{-1})	0.0003	–0.0005	0.0011	4.68E–01	0.0001	–0.0007	0.0008	8.59E–01
HDL VS TG (mmol·L^{-1})	0.0002	0.0000	0.0005	2.14E–02	0.0002	0.0000	0.0004	6.22E–02
Total TG (mmol·L^{-1})	0.0332	0.0040	0.0624	2.68E–02	0.0240	–0.0029	0.0508	7.89E–02
VLDL size (nm)	0.3324	0.1090	0.5558	4.25E–03	0.2612	0.0569	0.4656	1.32E–02
LDL size (nm)	–0.0087	–0.0217	0.0043	1.84E–01	–0.0073	–0.0211	0.0065	2.92E–01
HDL size (nm)	0.0100	–0.0013	0.0214	8.24E–02	0.0121	0.0009	0.0233	3.53E–02

Regression coefficients are in absolute concentration units of lipoprotein measures per SD unit increment of activity variable.
Model 1 is adjusted for baseline values of accelerometer wear time, age, lipoprotein measure, parents' education, sex, and sexual maturity. Cluster-robust standard errors were calculated, clustered on the school variable.
Model 2 is adjusted for baseline waist circumference in addition to the Model 1 covariates.
p values should be interpreted at a Bonferroni-corrected threshold of 0.01.
In notation of *p* values 1.23E–02 stands for "1.23 times 10 to the power of –02" or 0.0123.
Abbreviations: CI = confidence interval; CM = chylomicron; HDL = high-density lipoprotein; LDL = low-density lipoprotein; SD = standard deviation; VLDL = very low-density lipoprotein; -C = cholesterol; -L = large; -M = medium; -PN = particle number; -S = small; -TG = triglycerides; -VL = very large; -VS = very small.

Table 7. Associations between change in vigorous-intensity physical activity (VPA) and follow-up lipoprotein measures

Lipoprotein measure	Model 1				Model 2			
	Coefficient	Lower CI	Upper CI	*p* value	Coefficient	Lower CI	Upper CI	*p* value
CM PN (nmol·L^{-1})	0.0108	–0.0042	0.0258	1.53E–01	0.0140	–0.0018	0.0299	8.21E–02
VLDL L1 PN (nmol·L^{-1})	0.0235	–0.0249	0.0719	3.35E–01	0.0345	–0.0189	0.0879	2.01E–01
VLDL L2 PN (nmol·L^{-1})	0.0966	–0.0874	0.2806	2.97E–01	0.1375	–0.0677	0.3428	1.85E–01
VLDL L3 PN (nmol·L^{-1})	–0.0185	–0.5147	0.4776	9.41E–01	0.0546	–0.4946	0.6039	8.43E–01
VLDL M PN (nmol·L^{-1})	–0.1108	–0.9120	0.6903	7.83E–01	–0.0480	–0.9019	0.8058	9.11E–01
VLDL S PN (nmol·L^{-1})	–0.4840	–1.2367	0.2687	2.03E–01	–0.4723	–1.2328	0.2883	2.19E–01
LDL L PN (nmol·L^{-1})	–0.7025	–2.7500	1.3449	4.95E–01	–0.6772	–2.7441	1.3896	5.14E–01
LDL M PN (nmol·L^{-1})	0.4207	–3.8552	4.6965	8.44E–01	0.5066	–3.8003	4.8135	8.15E–01
LDL S PN (nmol·L^{-1})	0.3272	–2.0146	2.6690	7.81E–01	0.3961	–1.9682	2.7604	7.38E–01
LDL VS PN (nmol·1^{-1})	0.0164	–1.1083	1.1411	9.77E–01	0.0496	–1.0897	1.1888	9.31E–01
HDL VL PN (nmol·L^{-1})	0.4299	–4.8948	5.7546	8.72E–01	0.2333	–5.0853	5.5518	9.30E–01
HDL L PN (nmol·L^{-1})	6.6720	–33.3875	46.7316	7.40E–01	4.8238	–35.1531	44.8007	8.10E–01
HDL M PN (nmol·L^{-1})	–6.9981	–43.5765	29.5802	7.03E–01	–9.3789	–45.9249	27.1672	6.09E–01
HDL S PN (nmol·L^{-1})	–2.5932	–33.9902	28.8037	8.69E–01	–1.3061	–33.1582	30.5460	9.35E–01
HDL VS PN (nmol·L^{-1})	2.0747	–13.7713	17.9207	7.94E–01	2.9408	–13.2640	19.1457	7.18E–01
CM C (mmol·L^{-1})	0.0003	–0.0003	0.0009	2.95E–01	0.0004	–0.0002	0.0010	1.85E–01
VLDL C (mmol·L^{-1})	–0.0051	–0.0181	0.0079	4.36E–01	–0.0045	–0.0182	0.0091	5.07E–01
VLDL L1 C (mmol·L^{-1})	0.0001	–0.0006	0.0008	7.62E–01	0.0003	–0.0006	0.0011	5.37E–01
VLDL L2 C (mmol·L^{-1})	0.0004	–0.0015	0.0022	6.69E–01	0.0007	–0.0013	0.0028	4.83E–01
VLDL L3 C (mmol·L^{-1})	–0.0014	–0.0054	0.0027	5.11E–01	–0.0010	–0.0055	0.0034	6.42E–01
VLDL M C (mmol·L^{-1})	–0.0018	–0.0068	0.0031	4.60E–01	–0.0016	–0.0067	0.0034	5.19E–01
VLDL S C (mmol·L^{-1})	–0.0018	–0.0051	0.0015	2.81E–01	–0.0018	–0.0051	0.0016	2.89E–01
LDL C (mmol·L^{-1})	0.0014	–0.0202	0.0231	8.97E–01	0.0018	–0.0201	0.0237	8.69E–01
LDL L C (mmol·L^{-1})	–0.0021	–0.0098	0.0056	5.90E–01	–0.0020	–0.0097	0.0058	6.09E–01
LDL M C (mmol·L^{-1})	0.0010	–0.0092	0.0111	8.51E–01	0.0012	–0.0091	0.0114	8.21E–01
LDL S C (mmol·L^{-1})	0.0008	–0.0036	0.0052	7.18E–01	0.0009	–0.0035	0.0054	6.79E–01
LDL VS C (mmol·L^{-1})	0.0001	–0.0016	0.0017	9.36E–01	0.0001	–0.0015	0.0018	8.93E–01
HDL C (mmol·L^{-1})	0.0022	–0.0141	0.0184	7.89E–01	0.0012	–0.0150	0.0174	8.80E–01
HDL VL C (mmol·L^{-1})	0.0001	–0.0018	0.0021	8.90E–01	0.0001	–0.0019	0.0020	9.56E–01
HDL L C (mmol·L^{-1})	0.0014	–0.0071	0.0099	7.44E–01	0.0010	–0.0075	0.0095	8.18E–01
HDL M C (mmol·L^{-1})	–0.0006	–0.0056	0.0045	8.23E–01	–0.0010	–0.0061	0.0041	7.07E–01
HDL S C (mmol·L^{-1})	–0.0001	–0.0025	0.0022	9.14E–01	–0.0001	–0.0024	0.0023	9.58E–01

HDL VS C (mmol·L^{-1})	0.0001	–0.0008	0.0009	8.53E–01	0.0001	–0.0007	0.0010	7.96E–01
Total C (mmol·L^{-1})	–0.0034	–0.0337	0.0270	8.25E–01	–0.0030	–0.0337	0.0276	8.44E–01
Non-HDL C (mmol·L^{-1})	–0.0031	–0.0273	0.0212	8.01E–01	–0.0026	–0.0276	0.0223	8.32E–01
CM TG (mmol·L^{-1})	0.0012	–0.0005	0.0029	1.78E–01	0.0015	–0.0003	0.0033	9.78E–02
VLDL TG (mmol·L^{-1})	0.0026	–0.0113	0.0166	7.08E–01	0.0053	–0.0103	0.0209	4.98E–01
VLDL L1 TG (mmol·L^{-1})	0.0012	–0.0008	0.0032	2.22E–01	0.0017	–0.0005	0.0039	1.25E–01
VLDL L2 TG (mmol·L^{-1})	0.0021	–0.0019	0.0062	3.01E–01	0.0031	–0.0015	0.0076	1.83E–01
VLDL L3 TG (mmol·L^{-1})	0.0009	–0.0042	0.0060	7.19E–01	0.0019	–0.0038	0.0076	5.08E–01
VLDL M TG (mmol·L^{-1})	–0.0004	–0.0032	0.0025	8.07E–01	0.0000	–0.0031	0.0031	9.89E–01
VLDL S TG (mmol·L^{-1})	–0.0007	–0.0017	0.0004	2.01E–01	–0.0006	–0.0017	0.0004	2.42E–01
LDL TG (mmol·L^{-1})	–0.0013	–0.0033	0.0008	2.15E–01	–0.0012	–0.0033	0.0008	2.41E–01
LDL L TG (mmol·L^{-1})	–0.0008	–0.0021	0.0004	1.89E–01	–0.0008	–0.0021	0.0005	2.07E–01
LDL M TG (mmol·L^{-1})	–0.0004	–0.0012	0.0004	3.48E–01	–0.0004	–0.0012	0.0005	3.65E–01
LDL S TG (mmol·L^{-1})	0.0001	–0.0002	0.0005	3.76E–01	0.0002	–0.0002	0.0005	3.00E–01
LDL VS TG (mmol·L^{-1})	0.0001	–0.0001	0.0002	3.83E–01	0.0001	–0.0001	0.0002	2.71E–01
HDL TG (mmol·L^{-1})	–0.0006	–0.0029	0.0017	6.12E–01	–0.0005	–0.0028	0.0019	6.75E–01
HDL VL TG (mmol·L^{-1})	0.0000	–0.0002	0.0001	6.69E–01	0.0000	–0.0002	0.0001	6.30E–01
HDL L TG (mmol·L^{-1})	–0.0002	–0.0009	0.0004	5.23E–01	–0.0002	–0.0009	0.0004	4.55E–01
HDL M TG (mmol·L^{-1})	–0.0002	–0.0012	0.0007	6.09E–01	–0.0002	–0.0012	0.0008	7.02E–01
HDL S TG (mmol·L^{-1})	–0.0001	–0.0007	0.0005	7.60E–01	0.0000	–0.0006	0.0006	9.27E–01
HDL VS TG (mmol·L^{-1})	0.0000	–0.0001	0.0002	4.81E–01	0.0001	–0.0001	0.0002	2.90E–01
Total TG (mmol·L^{-1})	0.0034	–0.0141	0.0210	6.98E–01	0.0066	–0.0129	0.0261	4.98E–01
VLDL size (nm)	0.0876	–0.0400	0.2152	1.74E–01	0.1185	–0.0210	0.2580	9.45E–02
LDL size (nm)	–0.0062	–0.0166	0.0042	2.39E–01	–0.0068	–0.0172	0.0035	1.92E–01
HDL size (nm)	0.0005	–0.0098	0.0108	9.20E–01	–0.0001	–0.0104	0.0102	9.82E–01

Regression coefficients are in absolute concentration units of lipoprotein measures per SD unit increment of change in activity variable (follow-up minus baseline).
Model 1 is adjusted for change in accelerometer wear time, and baseline values of age, lipoprotein measure, parents' education, sex, and sexual maturity. Cluster-robust standard errors were calculated, clustered on the school variable.
Model 2 is adjusted for baseline waist circumference in addition to the Model 1 covariates.
p values should be interpreted at a Bonferroni-corrected threshold of 0.01.
In notation of *p* values 1.23E–02 stands for "1.23 times 10 to the power of –02" or 0.0123.
Abbreviations: CI = confidence interval; CM = chylomicron; HDL = high-density lipoprotein; LDL = low-density lipoprotein; SD = standard deviation; VLDL = very low-density lipoprotein; -C = cholesterol; -L = large; -M = medium; -PN = particle number; -S = small; -TG = triglycerides; -VL = very large; -VS = very small.

Table 8. Associations between change in moderate-intensity physical activity (MPA) and follow-up lipoprotein measures

Lipoprotein measure	Model 1				Model 2			
	Coefficient	Lower CI	Upper CI	*p* value	Coefficient	Lower CI	Upper CI	*p* value
CM PN (nmol·L^{-1})	0.0106	–0.0157	0.0369	4.22E–01	0.0145	–0.0114	0.0405	2.67E–01
VLDL L1 PN (nmol·L^{-1})	0.0111	–0.0692	0.0914	7.82E–01	0.0254	–0.0542	0.1051	5.25E–01
VLDL L2 PN (nmol·L^{-1})	0.0350	–0.2491	0.3192	8.06E–01	0.0881	–0.1937	0.3699	5.34E–01
VLDL L3 PN (nmol·L^{-1})	–0.2306	–0.8695	0.4083	4.73E–01	–0.1223	–0.7639	0.5193	7.04E–01
VLDL M PN (nmol·L^{-1})	–0.5630	–1.3596	0.2336	1.62E–01	–0.4670	–1.2577	0.3237	2.42E–01
VLDL S PN (nmol·L^{-1})	–0.9850	–1.6634	–0.3065	5.20E–03	–0.9667	–1.6438	–0.2896	5.94E–03
LDL L PN (nmol·L^{-1})	–1.1913	–3.6290	1.2463	3.32E–01	–1.1550	–3.5964	1.2865	3.47E–01
LDL M PN (nmol·L^{-1})	2.5004	–3.3957	8.3966	3.99E–01	2.6286	–3.2097	8.4669	3.71E–01
LDL S PN (nmol·L^{-1})	1.8869	–1.1943	4.9681	2.25E–01	1.9861	–1.0460	5.0182	1.95E–01
LDL VS PN (nmol·1^{-1})	0.5127	–0.9877	2.0130	4.96E–01	0.5580	–0.9190	2.0351	4.52E–01
HDL VL PN (nmol·L^{-1})	–0.7404	–6.0243	4.5436	7.80E–01	–1.0911	–6.3625	4.1802	6.80E–01
HDL L PN (nmol·L^{-1})	2.1702	–37.5730	41.9133	9.13E–01	–0.9257	–40.6145	38.7632	9.63E–01
HDL M PN (nmol·L^{-1})	–20.0602	–57.0219	16.9015	2.82E–01	–23.0438	–60.2602	14.1726	2.20E–01
HDL S PN (nmol·L^{-1})	4.4812	–34.2539	43.2162	8.18E–01	6.1830	–32.6127	44.9787	7.51E–01
HDL VS PN (nmol·L^{-1})	10.9407	–6.8116	28.6931	2.22E–01	11.9216	–5.5045	29.3477	1.76E–01
CM C (mmol·L^{-1})	0.0001	–0.0009	0.0011	8.40E–01	0.0002	–0.0007	0.0012	6.17E–01
VLDL C (mmol·L^{-1})	–0.0138	–0.0265	–0.0011	3.41E–02	–0.0127	–0.0256	0.0002	5.35E–02
VLDL L1 C (mmol·L^{-1})	–0.0001	–0.0013	0.0010	7.95E–01	0.0000	–0.0011	0.0012	9.34E–01
VLDL L2 C (mmol·L^{-1})	–0.0005	–0.0032	0.0022	7.17E–01	–0.0001	–0.0028	0.0026	9.66E–01
VLDL L3 C (mmol·L^{-1})	–0.0031	–0.0078	0.0015	1.78E–01	–0.0027	–0.0073	0.0020	2.62E–01
VLDL M C (mmol·L^{-1})	–0.0043	–0.0077	–0.0009	1.36E–02	–0.0040	–0.0074	–0.0005	2.42E–02
VLDL S C (mmol·L^{-1})	–0.0040	–0.0071	–0.0009	1.12E–02	–0.0040	–0.0070	–0.0009	1.19E–02
LDL C (mmol·L^{-1})	0.0112	–0.0172	0.0395	4.33E–01	0.0118	–0.0163	0.0399	4.03E–01
LDL L C (mmol·L^{-1})	–0.0023	–0.0113	0.0067	6.08E–01	–0.0022	–0.0112	0.0068	6.24E–01
LDL M C (mmol·L^{-1})	0.0071	–0.0064	0.0205	2.96E–01	0.0074	–0.0060	0.0207	2.73E–01
LDL S C (mmol·L^{-1})	0.0041	–0.0017	0.0098	1.60E–01	0.0042	–0.0014	0.0098	1.35E–01
LDL VS C (mmol·L^{-1})	0.0010	–0.0011	0.0032	3.34E–01	0.0011	–0.0010	0.0032	2.95E–01
HDL C (mmol·L^{-1})	0.0006	–0.0149	0.0160	9.40E–01	–0.0010	–0.0166	0.0147	9.03E–01
HDL VL C (mmol·L^{-1})	–0.0004	–0.0024	0.0016	6.87E–01	–0.0006	–0.0026	0.0014	5.81E–01
HDL L C (mmol·L^{-1})	–0.0007	–0.0097	0.0083	8.71E–01	–0.0015	–0.0105	0.0076	7.45E–01
HDL M C (mmol·L^{-1})	–0.0010	–0.0059	0.0039	6.71E–01	–0.0015	–0.0065	0.0034	5.36E–01
HDL S C (mmol·L^{-1})	0.0010	–0.0018	0.0037	4.81E–01	0.0011	–0.0017	0.0038	4.43E–01

HDL VS C (mmol·L^{-1})	0.0006	–0.0004	0.0015	2.20E–01	0.0006	–0.0003	0.0015	1.85E–01
Total C (mmol·L^{-1})	–0.0079	–0.0445	0.0286	6.65E–01	–0.0075	–0.0440	0.0290	6.82E–01
Non-HDL C (mmol·L^{-1})	–0.0072	–0.0387	0.0243	6.49E–01	–0.0063	–0.0379	0.0253	6.89E–01
CM TG (mmol·L^{-1})	0.0012	–0.0019	0.0042	4.51E–01	0.0016	–0.0014	0.0046	2.96E–01
VLDL TG (mmol·L^{-1})	–0.0032	–0.0237	0.0173	7.54E–01	0.0004	–0.0199	0.0207	9.66E–01
VLDL L1 TG (mmol·L^{-1})	0.0008	–0.0027	0.0044	6.45E–01	0.0014	–0.0021	0.0049	4.23E–01
VLDL L2 TG (mmol·L^{-1})	0.0008	–0.0058	0.0075	8.00E–01	0.0020	–0.0046	0.0087	5.37E–01
VLDL L3 TG (mmol·L^{-1})	–0.0012	–0.0084	0.0061	7.47E–01	0.0002	–0.0070	0.0074	9.62E–01
VLDL M TG (mmol·L^{-1})	–0.0018	–0.0051	0.0014	2.60E–01	–0.0014	–0.0045	0.0018	3.92E–01
VLDL S TG (mmol·L^{-1})	–0.0012	–0.0021	–0.0003	8.12E–03	–0.0012	–0.0020	–0.0003	1.11E–02
LDL TG (mmol·L^{-1})	–0.0021	–0.0043	0.0001	6.42E–02	–0.0020	–0.0042	0.0002	7.42E–02
LDL L TG (mmol·L^{-1})	–0.0014	–0.0025	–0.0004	8.51E–03	–0.0014	–0.0024	–0.0003	1.04E–02
LDL M TG (mmol·L^{-1})	–0.0006	–0.0015	0.0003	1.75E–01	–0.0006	–0.0015	0.0003	1.85E–01
LDL S TG (mmol·L^{-1})	0.0001	–0.0004	0.0006	6.29E–01	0.0001	–0.0003	0.0006	5.40E–01
LDL VS TG (mmol·L^{-1})	0.0000	–0.0002	0.0003	7.42E–01	0.0001	–0.0002	0.0003	5.97E–01
HDL TG (mmol·L^{-1})	–0.0021	–0.0048	0.0005	1.12E–01	–0.0020	–0.0047	0.0006	1.31E–01
HDL VL TG (mmol·L^{-1})	–0.0002	–0.0004	0.0000	8.13E–02	–0.0002	–0.0004	0.0000	7.83E–02
HDL L TG (mmol·L^{-1})	–0.0008	–0.0015	0.0000	4.85E–02	–0.0008	–0.0016	0.0000	3.81E–02
HDL M TG (mmol·L^{-1})	–0.0007	–0.0018	0.0003	1.75E–01	–0.0007	–0.0017	0.0004	2.15E–01
HDL S TG (mmol·L^{-1})	–0.0003	–0.0010	0.0004	4.59E–01	–0.0002	–0.0008	0.0005	6.21E–01
HDL VS TG (mmol·L^{-1})	0.0000	–0.0002	0.0002	7.61E–01	0.0001	–0.0001	0.0003	4.95E–01
Total TG (mmol·L^{-1})	–0.0044	–0.0315	0.0227	7.47E–01	–0.0001	–0.0269	0.0267	9.94E–01
VLDL size (nm)	0.0757	–0.1280	0.2793	4.60E–01	0.1134	–0.0892	0.3161	2.67E–01
LDL size (nm)	–0.0138	–0.0228	–0.0048	3.20E–03	–0.0144	–0.0233	–0.0056	1.92E–03
HDL size (nm)	–0.0038	–0.0154	0.0079	5.21E–01	–0.0048	–0.0165	0.0069	4.14E–01

Regression coefficients are in absolute concentration units of lipoprotein measures per SD unit increment of change in activity variable (follow-up minus baseline).
Model 1 is adjusted for change in accelerometer wear time, and baseline values of age, lipoprotein measure, parents' education, sex, and sexual maturity. Cluster-robust standard errors were calculated, clustered on the school variable.
Model 2 is adjusted for baseline waist circumference in addition to the Model 1 covariates.
p values should be interpreted at a Bonferroni-corrected threshold of 0.01.
In notation of *p* values 1.23E–02 stands for "1.23 times 10 to the power of –02" or 0.0123.
Abbreviations: CI = confidence interval; CM = chylomicron; HDL = high-density lipoprotein; LDL = low-density lipoprotein; SD = standard deviation; VLDL = very low-density lipoprotein; -C = cholesterol; -L = large; -M = medium; -PN = particle number; -S = small; -TG = triglycerides; -VL = very large; -VS = very small.

Table 9. Associations between change in light-intensity physical activity (LPA) and follow-up lipoprotein measures

Lipoprotein measure	Model 1				Model 2			
	Coefficient	Lower CI	Upper CI	*p* value	Coefficient	Lower CI	Upper CI	*p* value
CM PN (nmol·L^{-1})	0.0154	–0.0195	0.0503	3.82E–01	0.0211	–0.0135	0.0556	2.27E–01
VLDL L1 PN (nmol·L^{-1})	0.0163	–0.0842	0.1169	7.46E–01	0.0364	–0.0631	0.1360	4.66E–01
VLDL L2 PN (nmol·L^{-1})	0.0063	–0.3423	0.3549	9.71E–01	0.0813	–0.2627	0.4254	6.38E–01
VLDL L3 PN (nmol·L^{-1})	–0.2353	–0.9772	0.5067	5.28E–01	–0.0865	–0.8080	0.6350	8.11E–01
VLDL M PN (nmol·L^{-1})	–0.4388	–1.4030	0.5254	3.66E–01	–0.2843	–1.1942	0.6256	5.34E–01
VLDL S PN (nmol·L^{-1})	–0.3548	–1.1115	0.4019	3.52E–01	–0.3149	–1.0358	0.4060	3.85E–01
LDL L PN (nmol·L^{-1})	–0.5804	–2.9999	1.8390	6.33E–01	–0.4907	–2.8443	1.8628	6.78E–01
LDL M PN (nmol·L^{-1})	0.0889	–5.4470	5.6248	9.74E–01	0.4230	–5.1360	5.9820	8.79E–01
LDL S PN (nmol·L^{-1})	0.1158	–3.0951	3.3267	9.43E–01	0.3603	–2.8580	3.5785	8.23E–01
LDL VS PN (nmol·1^{-1})	0.0616	–1.4823	1.6055	9.37E–01	0.1714	–1.3817	1.7246	8.26E–01
HDL VL PN (nmol·L^{-1})	1.8640	–3.4811	7.2091	4.88E–01	1.3078	–3.8547	6.4703	6.14E–01
HDL L PN (nmol·L^{-1})	12.2894	–29.2411	53.8199	5.56E–01	7.8932	–32.6521	48.4384	6.98E–01
HDL M PN (nmol·L^{-1})	12.5207	–25.9716	51.0130	5.17E–01	7.4208	–31.4532	46.2948	7.04E–01
HDL S PN (nmol·L^{-1})	1.4009	–36.7522	39.5539	9.42E–01	4.5969	–33.2153	42.4090	8.08E–01
HDL VS PN (nmol·L^{-1})	–4.2003	–23.8743	15.4736	6.71E–01	–2.2451	–21.5280	17.0378	8.16E–01
CM C (mmol·L^{-1})	0.0004	–0.0009	0.0016	5.61E–01	0.0006	–0.0006	0.0018	3.46E–01
VLDL C (mmol·L^{-1})	0.0007	–0.0142	0.0156	9.22E–01	0.0024	–0.0121	0.0169	7.41E–01
VLDL L1 C (mmol·L^{-1})	0.0001	–0.0013	0.0014	9.25E–01	0.0003	–0.0010	0.0016	6.18E–01
VLDL L2 C (mmol·L^{-1})	–0.0001	–0.0033	0.0031	9.70E–01	0.0006	–0.0025	0.0037	7.12E–01
VLDL L3 C (mmol·L^{-1})	–0.0020	–0.0074	0.0033	4.53E–01	–0.0013	–0.0066	0.0041	6.39E–01
VLDL M C (mmol·L^{-1})	0.0008	–0.0038	0.0053	7.30E–01	0.0013	–0.0031	0.0057	5.51E–01
VLDL S C (mmol·L^{-1})	–0.0009	–0.0041	0.0023	5.63E–01	–0.0008	–0.0039	0.0022	5.80E–01
LDL C (mmol·L^{-1})	0.0016	–0.0243	0.0276	9.00E–01	0.0032	–0.0227	0.0291	8.05E–01
LDL L C (mmol·L^{-1})	–0.0030	–0.0119	0.0059	4.99E–01	–0.0028	–0.0114	0.0058	5.18E–01
LDL M C (mmol·L^{-1})	–0.0004	–0.0127	0.0119	9.44E–01	0.0003	–0.0119	0.0126	9.59E–01
LDL S C (mmol·L^{-1})	0.0004	–0.0053	0.0061	8.93E–01	0.0009	–0.0048	0.0065	7.64E–01
LDL VS C (mmol·L^{-1})	0.0001	–0.0020	0.0022	8.95E–01	0.0003	–0.0018	0.0024	7.72E–01
HDL C (mmol·L^{-1})	0.0063	–0.0086	0.0212	3.99E–01	0.0041	–0.0106	0.0188	5.80E–01
HDL VL C (mmol·L^{-1})	0.0009	–0.0010	0.0029	3.38E–01	0.0007	–0.0012	0.0026	4.50E–01
HDL L C (mmol·L^{-1})	0.0028	–0.0061	0.0117	5.29E–01	0.0018	–0.0069	0.0104	6.80E–01
HDL M C (mmol·L^{-1})	0.0030	–0.0016	0.0075	1.97E–01	0.0022	–0.0024	0.0067	3.44E–01
HDL S C (mmol·L^{-1})	–0.0002	–0.0028	0.0023	8.57E–01	–0.0001	–0.0026	0.0025	9.60E–01

HDL VS C (mmol·L^{-1})	–0.0002	–0.0012	0.0007	6.39E–01	–0.0002	–0.0011	0.0008	7.53E–01
Total C (mmol·L^{-1})	0.0084	–0.0259	0.0427	6.25E–01	0.0095	–0.0247	0.0437	5.79E–01
Non-HDL C (mmol·L^{-1})	0.0044	–0.0259	0.0348	7.70E–01	0.0064	–0.0239	0.0366	6.75E–01
CM TG (mmol·L^{-1})	0.0021	–0.0020	0.0061	3.09E–01	0.0027	–0.0013	0.0067	1.79E–01
VLDL TG (mmol·L^{-1})	–0.0021	–0.0264	0.0222	8.63E–01	0.0030	–0.0207	0.0267	8.00E–01
VLDL L1 TG (mmol·L^{-1})	0.0014	–0.0030	0.0058	5.33E–01	0.0022	–0.0021	0.0066	3.11E–01
VLDL L2 TG (mmol·L^{-1})	0.0008	–0.0072	0.0088	8.47E–01	0.0025	–0.0054	0.0104	5.31E–01
VLDL L3 TG (mmol·L^{-1})	–0.0016	–0.0101	0.0069	7.03E–01	0.0003	–0.0080	0.0085	9.52E–01
VLDL M TG (mmol·L^{-1})	–0.0021	–0.0059	0.0017	2.78E–01	–0.0014	–0.0050	0.0023	4.54E–01
VLDL S TG (mmol·L^{-1})	–0.0006	–0.0016	0.0005	2.84E–01	–0.0005	–0.0015	0.0006	3.71E–01
LDL TG (mmol·L^{-1})	0.0008	–0.0023	0.0038	6.24E–01	0.0009	–0.0022	0.0040	5.74E–01
LDL L TG (mmol·L^{-1})	–0.0002	–0.0014	0.0011	7.93E–01	–0.0001	–0.0013	0.0012	8.82E–01
LDL M TG (mmol·L^{-1})	0.0004	–0.0008	0.0015	5.36E–01	0.0004	–0.0008	0.0016	5.13E–01
LDL S TG (mmol·L^{-1})	0.0004	–0.0003	0.0010	2.46E–01	0.0004	–0.0002	0.0010	1.82E–01
LDL VS TG (mmol·L^{-1})	0.0002	–0.0002	0.0005	3.32E–01	0.0002	–0.0001	0.0005	2.22E–01
HDL TG (mmol·L^{-1})	0.0005	–0.0027	0.0038	7.42E–01	0.0008	–0.0024	0.0039	6.36E–01
HDL VL TG (mmol·L^{-1})	0.0001	–0.0001	0.0003	3.41E–01	0.0001	–0.0001	0.0003	4.03E–01
HDL L TG (mmol·L^{-1})	0.0003	–0.0005	0.0011	5.12E–01	0.0002	–0.0007	0.0010	6.67E–01
HDL M TG (mmol·L^{-1})	0.0001	–0.0012	0.0015	8.65E–01	0.0002	–0.0010	0.0015	7.07E–01
HDL S TG (mmol·L^{-1})	0.0001	–0.0008	0.0010	8.26E–01	0.0002	–0.0006	0.0010	5.89E–01
HDL VS TG (mmol·L^{-1})	0.0001	–0.0002	0.0003	6.62E–01	0.0001	–0.0001	0.0003	3.92E–01
Total TG (mmol·L^{-1})	0.0016	–0.0312	0.0343	9.25E–01	0.0077	–0.0241	0.0394	6.31E–01
VLDL size (nm)	0.0503	–0.2013	0.3019	6.90E–01	0.1026	–0.1473	0.3524	4.14E–01
LDL size (nm)	–0.0041	–0.0162	0.0081	5.04E–01	–0.0053	–0.0171	0.0065	3.73E–01
HDL size (nm)	0.0039	–0.0081	0.0158	5.21E–01	0.0024	–0.0092	0.0140	6.83E–01

Regression coefficients are in absolute concentration units of lipoprotein measures per SD unit increment of change in activity variable (follow-up minus baseline).
Model 1 is adjusted for change in accelerometer wear time, and baseline values of age, lipoprotein measure, parents' education, sex, and sexual maturity. Cluster-robust standard errors were calculated, clustered on the school variable.
Model 2 is adjusted for baseline waist circumference in addition to the Model 1 covariates.
p values should be interpreted at a Bonferroni-corrected threshold of 0.01.
In notation of *p* values 1.23E–02 stands for "1.23 times 10 to the power of –02" or 0.0123.
Abbreviations: CI = confidence interval; CM = chylomicron; HDL = high-density lipoprotein; LDL = low-density lipoprotein; SD = standard deviation; VLDL = very low-density lipoprotein; -C = cholesterol; -L = large; -M = medium; -PN = particle number; -S = small; -TG = triglycerides; -VL = very large; -VS = very small.

Table 10. Associations between change in sedentary time and follow-up lipoprotein measures

Lipoprotein measure	Model 1				Model 2			
	Coefficient	Lower CI	Upper CI	*p* value	Coefficient	Lower CI	Upper CI	*p* value
CM PN (nmol·L^{-1})	–0.0208	–0.0572	0.0156	2.57E–01	–0.0280	–0.0650	0.0089	1.34E–01
VLDL L1 PN (nmol·L^{-1})	–0.0278	–0.1329	0.0773	5.98E–01	–0.0533	–0.1609	0.0544	3.26E–01
VLDL L2 PN (nmol·L^{-1})	–0.0632	–0.4262	0.2999	7.29E–01	–0.1581	–0.5306	0.2144	3.99E–01
VLDL L3 PN (nmol·L^{-1})	0.2758	–0.5123	1.0640	4.86E–01	0.0910	–0.7109	0.8930	8.21E–01
VLDL M PN (nmol·L^{-1})	0.5925	–0.4723	1.6572	2.70E–01	0.4134	–0.6324	1.4593	4.32E–01
VLDL S PN (nmol·L^{-1})	0.8479	0.0131	1.6826	4.66E–02	0.8078	–0.0024	1.6181	5.07E–02
LDL L PN (nmol·L^{-1})	1.1970	–1.3815	3.7754	3.56E–01	1.1072	–1.4480	3.6623	3.89E–01
LDL M PN (nmol·L^{-1})	–1.1072	–7.2414	5.0271	7.19E–01	–1.4464	–7.6239	4.7311	6.41E–01
LDL S PN (nmol·L^{-1})	–0.8629	–4.4405	2.7146	6.31E–01	–1.1161	–4.7132	2.4809	5.37E–01
LDL VS PN (nmol·1^{-1})	–0.2108	–1.9278	1.5061	8.07E–01	–0.3254	–2.0565	1.4057	7.08E–01
HDL VL PN (nmol·L^{-1})	–1.2934	–7.6965	5.1097	6.87E–01	–0.6608	–6.8660	5.5444	8.32E–01
HDL L PN (nmol·L^{-1})	–12.8189	–60.9713	35.3335	5.96E–01	–7.5945	–54.1897	39.0006	7.45E–01
HDL M PN (nmol·L^{-1})	0.6975	–41.5661	42.9611	9.74E–01	6.7164	–35.3489	48.7818	7.50E–01
HDL S PN (nmol·L^{-1})	–2.0608	–41.7468	37.6252	9.18E–01	–5.6777	–45.8857	34.5303	7.78E–01
HDL VS PN (nmol·L^{-1})	–1.6689	–22.5853	19.2476	8.74E–01	–3.9137	–24.8108	16.9835	7.09E–01
CM C (mmol·L^{-1})	–0.0004	–0.0017	0.0008	4.86E–01	–0.0007	–0.0020	0.0006	2.76E–01
VLDL C (mmol·L^{-1})	0.0069	–0.0090	0.0228	3.87E–01	0.0050	–0.0109	0.0209	5.30E–01
VLDL L1 C (mmol·L^{-1})	0.0000	–0.0015	0.0014	9.52E–01	–0.0004	–0.0018	0.0011	6.03E–01
VLDL L2 C (mmol·L^{-1})	0.0000	–0.0034	0.0035	9.82E–01	–0.0008	–0.0042	0.0027	6.66E–01
VLDL L3 C (mmol·L^{-1})	0.0034	–0.0022	0.0090	2.24E–01	0.0025	–0.0032	0.0083	3.85E–01
VLDL M C (mmol·L^{-1})	0.0018	–0.0027	0.0064	4.18E–01	0.0012	–0.0032	0.0057	5.82E–01
VLDL S C (mmol·L^{-1})	0.0030	–0.0007	0.0067	1.14E–01	0.0029	–0.0007	0.0065	1.12E–01
LDL C (mmol·L^{-1})	–0.0056	–0.0346	0.0233	6.98E–01	–0.0072	–0.0364	0.0219	6.21E–01
LDL L C (mmol·L^{-1})	0.0041	–0.0055	0.0137	3.96E–01	0.0038	–0.0056	0.0133	4.17E–01
LDL M C (mmol·L^{-1})	–0.0025	–0.0162	0.0112	7.14E–01	–0.0033	–0.0171	0.0105	6.34E–01
LDL S C (mmol·L^{-1})	–0.0020	–0.0084	0.0043	5.28E–01	–0.0025	–0.0089	0.0039	4.36E–01
LDL VS C (mmol·L^{-1})	–0.0005	–0.0028	0.0019	6.88E–01	–0.0006	–0.0030	0.0017	5.86E–01
HDL C (mmol·L^{-1})	–0.0060	–0.0238	0.0118	5.04E–01	–0.0033	–0.0208	0.0141	7.03E–01
HDL VL C (mmol·L^{-1})	–0.0006	–0.0030	0.0018	6.03E–01	–0.0004	–0.0027	0.0019	7.57E–01
HDL L C (mmol·L^{-1})	–0.0025	–0.0133	0.0084	6.50E–01	–0.0012	–0.0118	0.0093	8.13E–01
HDL M C (mmol·L^{-1})	–0.0016	–0.0067	0.0035	5.24E–01	–0.0007	–0.0057	0.0044	7.89E–01
HDL S C (mmol·L^{-1})	–0.0001	–0.0029	0.0027	9.20E–01	–0.0003	–0.0031	0.0025	8.16E–01

HDL VS C (mmol·L^{-1})	–0.0001	–0.0012	0.0010	9.08E–01	–0.0002	–0.0012	0.0009	7.82E–01
Total C (mmol·L^{-1})	–0.0015	–0.0415	0.0385	9.39E–01	–0.0027	–0.0430	0.0376	8.95E–01
Non-HDL C (mmol·L^{-1})	0.0011	–0.0322	0.0344	9.48E–01	–0.0008	–0.0347	0.0330	9.61E–01
CM TG (mmol·L^{-1})	–0.0026	–0.0068	0.0016	2.27E–01	–0.0034	–0.0076	0.0009	1.18E–01
VLDL TG (mmol·L^{-1})	0.0015	–0.0239	0.0269	9.07E–01	–0.0049	–0.0308	0.0210	7.05E–01
VLDL L1 TG (mmol·L^{-1})	–0.0019	–0.0065	0.0027	4.02E–01	–0.0030	–0.0077	0.0017	2.05E–01
VLDL L2 TG (mmol·L^{-1})	–0.0019	–0.0103	0.0065	6.51E–01	–0.0041	–0.0127	0.0046	3.49E–01
VLDL L3 TG (mmol·L^{-1})	0.0012	–0.0077	0.0101	7.82E–01	–0.0011	–0.0102	0.0080	8.06E–01
VLDL M TG (mmol·L^{-1})	0.0024	–0.0017	0.0065	2.42E–01	0.0015	–0.0025	0.0056	4.48E–01
VLDL S TG (mmol·L^{-1})	0.0012	0.0000	0.0023	4.60E–02	0.0011	–0.0001	0.0022	6.81E–02
LDL TG (mmol·L^{-1})	0.0007	–0.0026	0.0041	6.63E–01	0.0006	–0.0028	0.0040	7.23E–01
LDL L TG (mmol·L^{-1})	0.0010	–0.0004	0.0024	1.56E–01	0.0009	–0.0005	0.0024	1.89E–01
LDL M TG (mmol·L^{-1})	0.0001	–0.0012	0.0014	8.60E–01	0.0001	–0.0013	0.0014	8.95E–01
LDL S TG (mmol·L^{-1})	–0.0004	–0.0010	0.0003	2.44E–01	–0.0005	–0.0011	0.0002	1.80E–01
LDL VS TG (mmol·L^{-1})	–0.0002	–0.0005	0.0002	3.30E–01	–0.0002	–0.0006	0.0001	2.15E–01
HDL TG (mmol·L^{-1})	0.0006	–0.0030	0.0042	7.39E–01	0.0004	–0.0032	0.0039	8.42E–01
HDL VL TG (mmol·L^{-1})	0.0000	–0.0002	0.0002	9.96E–01	0.0000	–0.0002	0.0003	9.26E–01
HDL L TG (mmol·L^{-1})	0.0002	–0.0008	0.0011	7.15E–01	0.0003	–0.0007	0.0012	5.69E–01
HDL M TG (mmol·L^{-1})	0.0003	–0.0012	0.0017	7.12E–01	0.0001	–0.0013	0.0015	8.64E–01
HDL S TG (mmol·L^{-1})	0.0001	–0.0009	0.0010	9.12E–01	–0.0001	–0.0010	0.0008	8.17E–01
HDL VS TG (mmol·L^{-1})	–0.0001	–0.0003	0.0002	5.78E–01	–0.0001	–0.0004	0.0001	3.00E–01
Total TG (mmol·L^{-1})	–0.0013	–0.0358	0.0331	9.39E–01	–0.0090	–0.0438	0.0258	6.07E–01
VLDL size (nm)	–0.1062	–0.3673	0.1549	4.19E–01	–0.1738	–0.4433	0.0956	2.02E–01
LDL size (nm)	0.0107	–0.0029	0.0243	1.20E–01	0.0122	–0.0011	0.0254	7.17E–02
HDL size (nm)	–0.0017	–0.0154	0.0119	8.00E–01	0.0000	–0.0131	0.0132	9.95E–01

Regression coefficients are in absolute concentration units of lipoprotein measures per SD unit increment of change in activity variable (follow-up minus baseline).
Model 1 is adjusted for change in accelerometer wear time, and baseline values of age, lipoprotein measure, parents' education, sex, and sexual maturity. Cluster-robust standard errors were calculated, clustered on the school variable.
Model 2 is adjusted for baseline waist circumference in addition to the Model 1 covariates.
p values should be interpreted at a Bonferroni-corrected threshold of 0.01.
In notation of *p* values 1.23E–02 stands for "1.23 times 10 to the power of –02" or 0.0123.
Abbreviations: CI = confidence interval; CM = chylomicron; HDL = high-density lipoprotein; LDL = low-density lipoprotein; SD = standard deviation; VLDL = very low-density lipoprotein; -C = cholesterol; -L = large; -M = medium; -PN = particle number; -S = small; -TG = triglycerides; -VL = very large; -VS = very small.
